# Role of the mitochondrial protein cyclophilin D in skin wound healing and collagen secretion

**DOI:** 10.1172/jci.insight.169213

**Published:** 2024-04-02

**Authors:** Ritu Bansal, Monica Torres, Matthew Hunt, Nuoqi Wang, Margarita Chatzopoulou, Mansi Manchanda, Evan P. Taddeo, Cynthia Shu, Orian S. Shirihai, Etty Bachar-Wikstrom, Jakob D. Wikstrom

**Affiliations:** 1 Dermatology and Venereology Division, Department of Medicine (Solna), Karolinska Institutet, Stockholm, Sweden.; 2 Dermato-Venereology Clinic, Karolinska University Hospital, Stockholm, Sweden.; 3 Metabolism Theme,; 4 Department of Molecular and Medical Pharmacology, and; 5 Department of Medicine, Division of Endocrinology, David Geffen School of Medicine, UCLA, Los Angeles, California, USA.

**Keywords:** Dermatology, Collagens, Mitochondria, Skin

## Abstract

Central for wound healing is the formation of granulation tissue, which largely consists of collagen and whose importance stretches past wound healing, including being implicated in both fibrosis and skin aging. Cyclophilin D (CyD) is a mitochondrial protein that regulates the permeability transition pore, known for its role in apoptosis and ischemia-reperfusion. To date, the role of CyD in human wound healing and collagen generation has been largely unexplored. Here, we show that CyD was upregulated in normal wounds and venous ulcers, likely adaptive as CyD inhibition impaired reepithelialization, granulation tissue formation, and wound closure in both human and pig models. Overexpression of CyD increased keratinocyte migration and fibroblast proliferation, while its inhibition reduced migration. Independent of wound healing, CyD inhibition in fibroblasts reduced collagen secretion and caused endoplasmic reticulum collagen accumulation, while its overexpression increased collagen secretion. This was confirmed in a *Ppif*-KO mouse model, which showed a reduction in skin collagen. Overall, this study revealed previously unreported roles of CyD in skin, with implications for wound healing and beyond.

## Abstract 2

The mitochondrial protein cyclophilin D contributes to human skin wound healing by affecting granulation tissue formation and collagen secretion and deposition.

## Introduction

Normal skin wound healing is characterized by 4 overlapping phases: hemostasis, inflammation, proliferation, and tissue remodeling. Timely and regulated execution of these processes results in effective wound closure, while impairments result in chronic ulcerations in which venous leg ulcers are the most common (1–4). Essential for wound closure is reepithelialization by keratinocytes as well as extracellular matrix (ECM) deposition by dermal fibroblasts, which together form the granulation tissue that replaces lost tissue and serve as a scaffold for migrating and proliferating cells (3, 5, 6). In fact, many wound healing dressings contain collagen, the most abundant protein in the ECM as well as the most abundant protein overall in most organisms (7). Wound-related costs are estimated to be 2%–4% of healthcare budgets in the industrialized world, and this is expected to increase with population aging (8). Furthermore, recent decades of wound healing research have substantially increased the understanding of wound healing on different levels; however, it has largely failed to translate into widely used prohealing treatments, and thus new research initiatives are needed.

Since wounds at large are hypoxic, mitochondria have recently gained attention in wound healing because of their role as oxygen consumers during ATP production (9–12). ATP production was shown to be essential for wound healing processes such as angiogenesis, keratinocyte migration, differentiation, fibroblast proliferation, and collagen synthesis (11). However, the molecular understanding of how mitochondria impact central wound healing processes is still at its infancy (13). Understanding how key mitochondrial proteins affect wound healing could, thus, translate into novel therapeutic strategies.

Cyclophilin D (CyD) is a mitochondrial matrix peptidyl-prolyl *cis-trans* isomerase, the only mitochondrial member of the cyclophilin family, and is encoded by the *PPIF* gene. CyD is a well-known regulator of the mitochondrial permeability transition pore (mPTP), a multicomponent pore located in the inner mitochondrial membrane and known foremost for its role in apoptosis as well as for being 1 of 2 targets for the widely used immunosuppressant drug cyclosporine A (14, 15). CyD plays roles in various processes important to wound healing and the complex wound environment. Namely, CyD participates in the regulation of mitochondrial ATP production (16), which is vital for cell proliferation during wound repair. Additionally, CyD also contributes to maintaining appropriate levels of reactive oxygen species (ROS) (17), which are often elevated in the wound environment, and — in the case of excessive production and subsequent oxidative stress — can lead to chronic wound formation. Furthermore, CyD is involved in apoptosis, a process essential for balancing cell proliferation and resolving inflammation during normal wound healing. This role becomes increasingly substantial in hypoxic wounds, where abnormal increases in apoptosis are observed (13, 18). In addition, emerging data are pointing toward a role for some cyclophilins in ECM dynamics, including isomerization or folding of collagen (19, 20), such as in a recent study that showed that deletion of CyD prevented renal fibrosis (21). Thus, there are several reasons for investigating how CyD may affect wound healing, which to date is largely unexplored.

Herein, we studied the role of CyD in wound healing by utilizing a combination of human, porcine, and mouse models, as well as in vitro experiments, and discovered noncanonical roles for CyD in wound healing as well as in collagen synthesis and secretion.

## Results

### CyD is upregulated in the reepithelialization and granulation phase of normal acute human wound healing and chronic venous leg ulcers.

**Figure 1:**
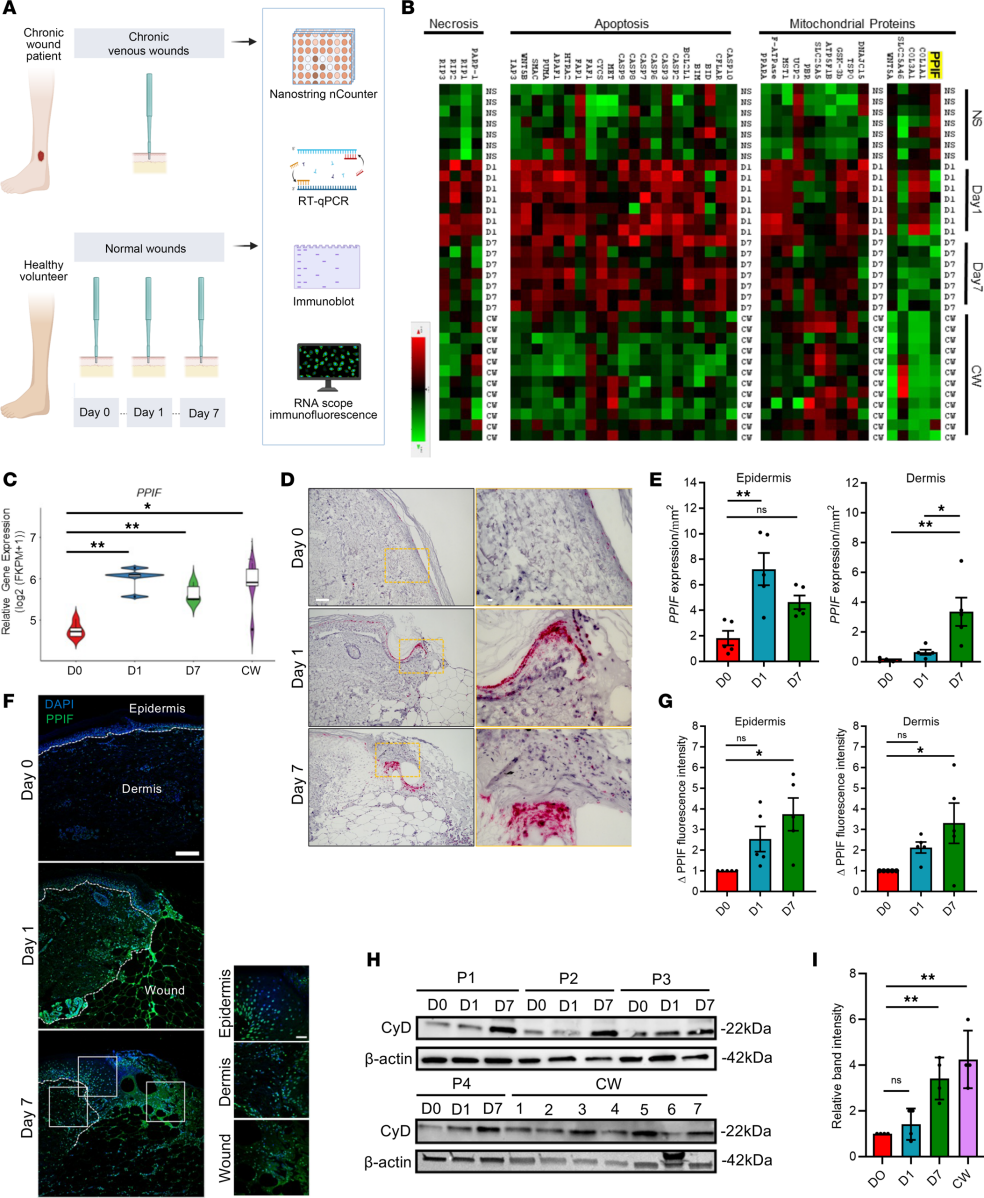
Cyclophilin D is upregulated in normal wound healing as well as in chronic venous leg ulcers in the elderly. (**A**) Summary of experimental procedures. (**B**) NanoString profiling showing expression of apoptosis, necrosis, and permeability transition pore-associated genes, including *PPIF* and its interactors. Heatmap illustrates alterations in gene expression with fold change > 1.5 (green, increased expression; red, reduced expression). *n* = 7 (day 0/1/7); 12 (CW). (**C**) Publicly available RNA-Seq gene expression analysis of *PPIF* in intact skin, wound, and CW biopsies. (**D** and **E**) Representative images and quantification (mean ± SEM) of *PPIF* RNA expression in wound healing days 0, 1, and 7 after in situ hybridization staining. *PPIF* RNA puncti are visible as pink dots. Scale bar: 100 μm (full size); 50 μm (zoom). *n* = 5. (**F**) Representative fluorescence images of day 0, 1, and 7 skin biopsies demonstrating the assessment of PPIF expression in human wound healing. The white lines depict the demarcation of the epidermis, dermis, and wound bed. Scale bar: 100 μm (full size); 50 μm (zoom). *n* = 5. (**G**) Quantification (mean ± SEM) of PPIF fluorescence intensity in the epidermis and dermis after normalization to day 0. (**H** and **I**) Immunoblot and quantification (mean ± SEM) showing CyD protein expression in human wound samples. *n* = 4. **P < 0.01; *P < 0.05; 2-way ANOVA.

To investigate the role of the mitochondrial protein CyD in normal acute human wound healing and the most common chronic wound (CW) type, venous leg ulcers, we performed NanoString (22) gene expression analysis, quantitative PCR (qPCR), RNAscope, immunofluorescence, and immunoblotting on human biopsy tissue from elderly volunteers and patients with CWs (Figure 1A). NanoString data demonstrate an upregulation of *PPIF* (1.8-fold change, *P* < 0.05) in healthy volunteers at day 7 following normal acute wound induction, as well as in CW skin (Figure 1B). As predicted from previous literature, apoptosis and necrosis genes were upregulated in normal acute wound healing (day 7); however, they decreased in chronic venous leg ulcers, opposite to the pattern shown for *PPIF*. This suggests that the role of CyD is not limited to cell death by apoptosis or necrosis and that CyD may be important for normal healthy wound healing (Figure 1B). Supporting this notion, we observed an increased genetic expression of the mPTP protein, *SLC25A5*, in normal wound healing. However, no change was observed in CW tissue (Figure 1B). In line with this observation, publicly available bulk RNA-Seq gene expression data (23) from similar biopsy material also show increased expression of *PPIF* (Figure 1C); *SLC25A5*, *SLC25A51*, *STAT3*, and *VDAC1* in normal skin wound healing; and decreased expression of these genes, except for *PPIF*, in CW tissue (Supplemental Figure 1, A–E; supplemental material available online with this article; https://doi.org/10.1172/jci.insight.169213DS1) (GSE174661, GSE196773). RNA in situ hybridization analysis further shows an increase in *PPIF* RNA expression in healthy volunteers at day 7 following normal acute wound induction (Figure 1, D and E).

To further validate these results, we performed qPCR on a separate set of RNA extracted from normal acute wounds and chronic venous ulcers. This experiment confirmed the upregulation of *PPIF* expression both in acute and venous ulcers as compared with healthy skin (2-fold change, *P* < 0.05) (Supplemental Figure 1F). To assess CyD expression at the protein level, we conducted immunofluorescence analysis of PPIF expression in wounded skin biopsies, which also confirmed the increased expression of CyD at the protein level (Figure 1, F and G). Additionally, immunoblot experiments on protein extracted from similar samples derived from the same donors as used in the NanoString experiment demonstrated a higher expression in both day 7 and chronic venous ulcer biopsies (Figure 1, H and I, and Supplemental Figure 1G). Protein expression of SIRT3 was not significantly different between wounded samples. Collectively, CyD was demonstrated to have a higher expression in both normal wound healing and chronic venous ulcers, suggesting a possible role in wound healing, albeit not necessarily coupled to its known effect on mPTP activity.

### Pharmacological CyD inhibition impairs reepithelialization and collagen deposition in human explant skin wound healing model.

**Figure 2:**
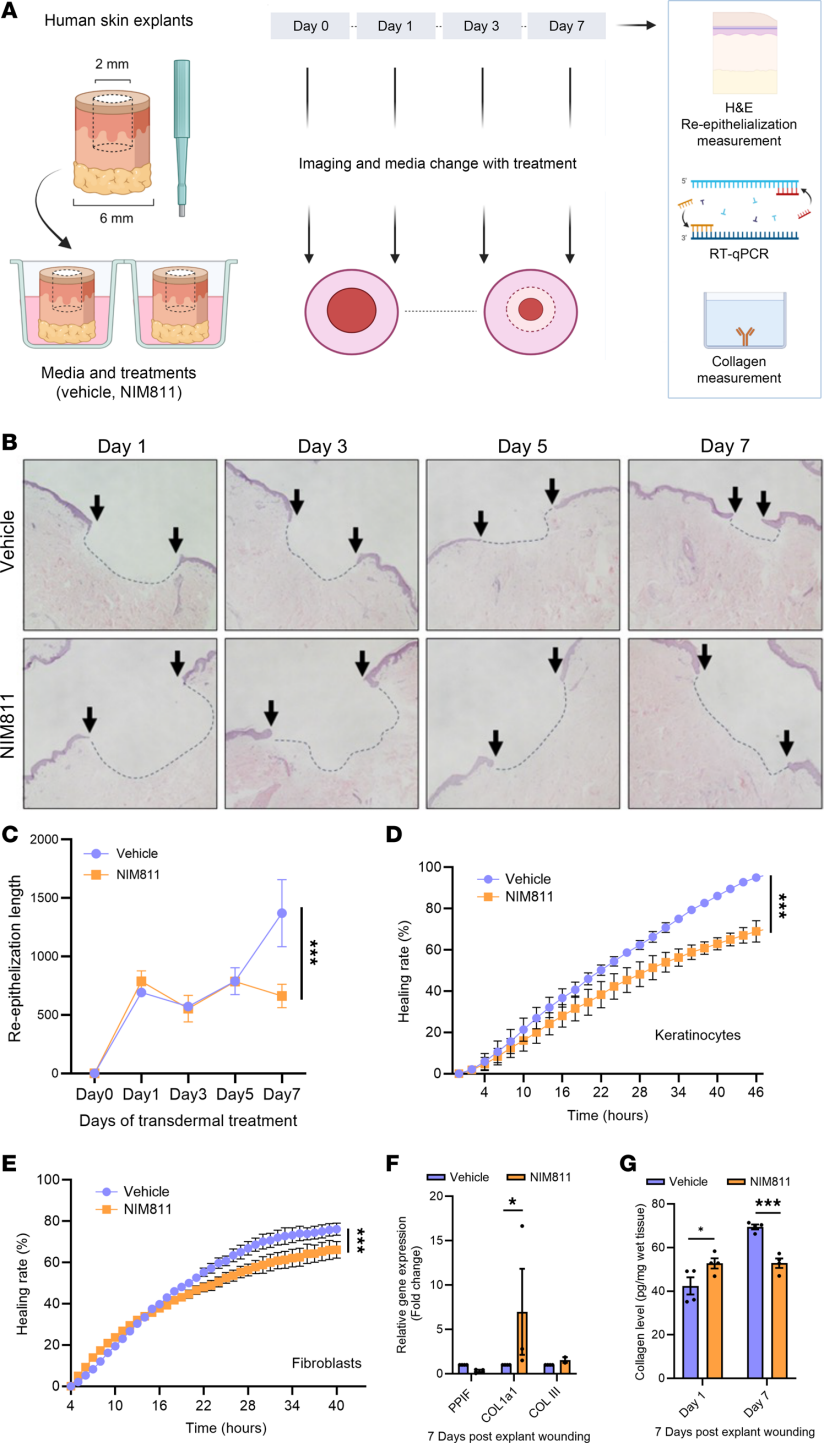
Cyclophilin D inhibition impairs human skin explant wound reepithelialization as well as in vitro scratch wound assay and alters collagen expression. (**A**) Illustration of treatments and experiments conducted. (**B** and **C**) H&E-stained sections and quantification (mean ± SEM) of explant skin wounds at different days after wounding as indicated after transdermal treatment with the specific CyD inhibitor NIM811. Arrows indicate wound size by marking epidermal edges. *n* =3. Magnification, ×4. (**D** and **E**) Scratch wound assay of keratinocytes and human primary fibroblasts treated with NIM811. (**F**) qPCR analysis (mean ± SEM) of *PPIF* (CyD) and *COL1A1* (collagen 1) expression in explants after transdermal treatment with NIM811 for 7 days. (**G**) Collagen level (mean ± SEM) by hydroxyproline assay after different days of NIM811 treatment. *n* = 3 for all experiments. ***P < 0.001; *P < 0.05.

To uncover the importance of CyD in both fibroblasts and keratinocytes, central in skin wound healing, we assessed the effect of its pharmacological inhibition with topical NIM811 treatment on the human ex vivo explant wound healing model, which is especially useful to study reepithelialization (24) (Figure 2A). NIM811 is a CyD inhibitor that is devoid of calcineurin inhibitory effects and, therefore, does not induce immunosuppression like cyclosporine A (25); it is often regarded as a specific CyD inhibitor, although it may also inhibit other nonmitochondrial cyclophilins to some degree. We, thus, applied NIM811 on the wounds and observed the rate of wound healing for 7 days using H&E staining. Here, we found that NIM811 decreased the wound healing rate (Figure 2, B and C) as well as reduced granulation tissue formation in the wounds (Supplemental Figure 2A). CyD inhibition by NIM811 treatment was confirmed by its reduction of PTP opening in the calcein-cobalt chloride quenching assay (Supplemental Figure 2, B and C), and we additionally ruled out the possibility that these effects were due to a reduction of fibroblast or keratinocyte cell viability (Supplemental Figure 2, D and E). To further corroborate the ex vivo explant wound results, we performed scratch assays, an in vitro wound healing assay that integrates both cell migration and proliferation, using keratinocytes and cultured primary human fibroblasts. Here, we found that NIM811 treatment resulted in a decrease in wound healing in both cell types (Figure 2, D and E).

Next, we used CyD inhibition by NIM811 treatment in order to assess the changes in granulation tissue formation, an important process in wound healing. Here, we measured its prime component collagen after 7 days of treatment in the ex vivo explant wound healing model. Unexpectedly, we observed an increase in collagen 1 gene expression through qPCR analysis (Figure 2F). Since collagen is ultimately secreted to the ECM, we measured its deposition using a hydroxyproline-based assay (26) and observed a decrease in collagen deposition at 7 days, despite increased gene expression (Figure 2G). Thus, CyD inhibition impaired both cell migration as well as collagen secretion and deposition.

### Pharmacological CyD inhibition impairs porcine wound closure and collagen deposition.

**Figure 3:**
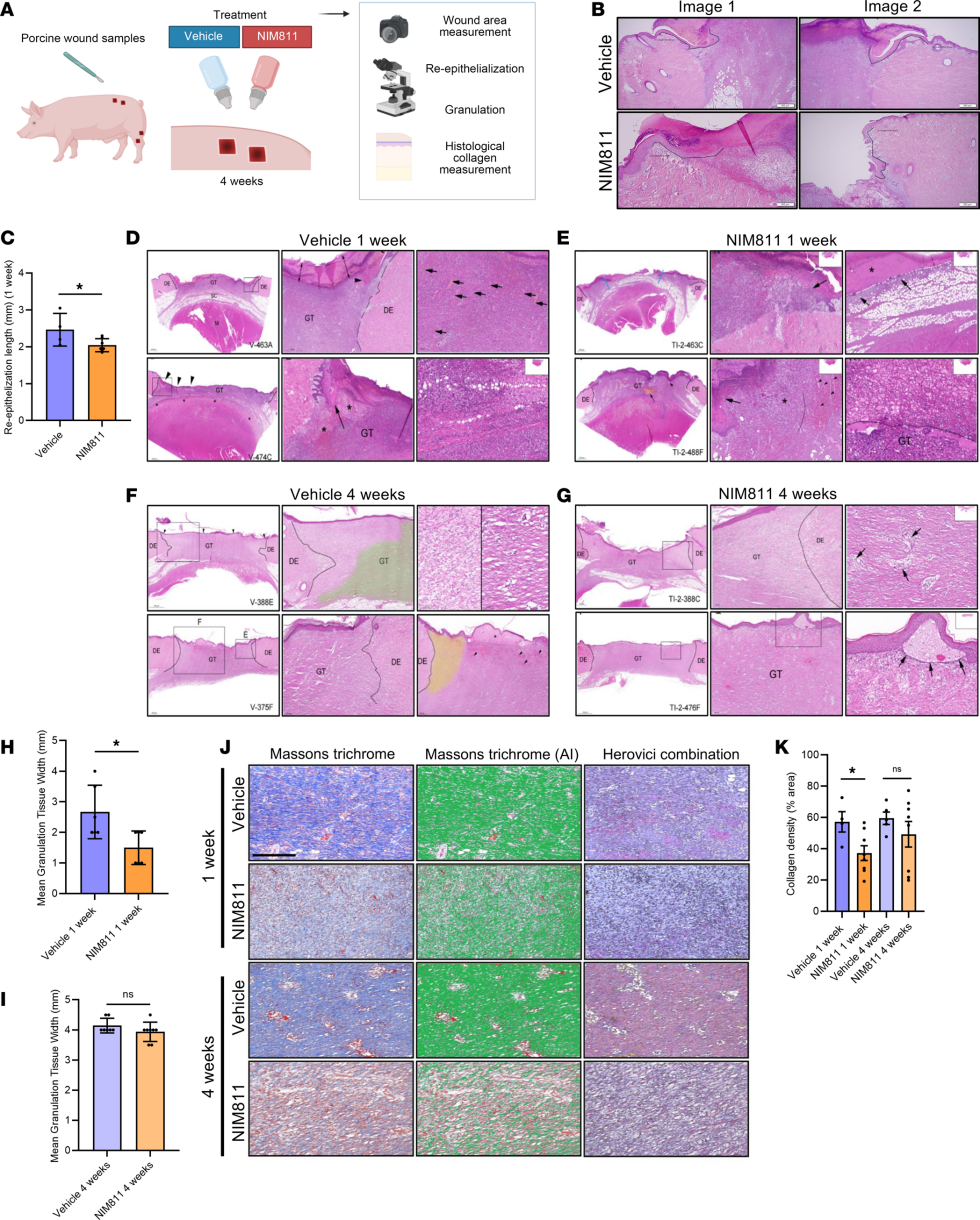
Cyclophilin D inhibition impairs porcine wound closure, granulation, and collagen formation. (**A**) Illustration of treatments and experiments conducted. (**B** and **C**) Representative H&E-stained sections (**B**) used for quantification (mean ± SEM) of reepithelialization on day 7 post wounding (**C**). The superimposed line highlights the neoepidermis. *n* = 4 pigs and 2 wounds per group (8 wounds per pig). (**D**–**G**) Representative H&E-stained sections for wound granulation assessments at day 7 and day 14 after the wound infliction and treatment vehicle (**D** and **F**) or NIM811 (**E** and **G**). The interface of the granulation tissue and the dermal edge is marked with a black line. Scars (granulation tissue) are covered by a thick fibrinopurulent crust (spanned by double-headed arrows). Fibroblasts are oriented perpendicular to blood vessels (arrows). The scar is located between 2 black lines on the right and left. DE, dermal edge; GT, granulation tissue; mod, moderate; RE, reepithelialization; S, scar; SC, s.c.; v, vehicle; Ti-2, NIM811 treatment. (**H** and **I**) Quantification (mean ± SEM) of mean granulation tissue width in vehicle- and NIM811-treated wounds at 1 week and 4 weeks. (**J**) Representative Masson’s trichrome– and Herovici combination–stained sections of porcine wounds to assess collagen density and mature versus newly formed collagen, respectively. Note that Masson’s trichrome stains collagen with blue color (unprocessed images/without AI) or green color (processed images/with AI). Herovici combination stains mature dense collagen red while and newly formed collagen as blue. Scale bar: 50 μm. (**K**) Histopathological scoring of collagen density. Two-tailed, unpaired *t* test. *n* = 3. *P < 0.05.

While there is no perfect animal model for human wound healing, due to the anatomical and physiological similarities of pig and human skin, pigs represent the most human-like wound healing model (27, 28). To examine how CyD affects wound healing in vivo, we treated full-thickness skin wounds in Göttingen mini pigs topically with NIM811 and measured healing rates, reepithelialization, and granulation tissue formation (Figure 3A). Reepithelialization was identified by the presence of a thin neoepidermis on the wound bed and granulation tissue by the presence of a typical mix of myofibroblasts, neovasculature, immune cell infiltrate — as well as fibroblasts and immature collagen deposition — as assessed by an experienced veterinarian pathologist (29). 

The data suggest an inhibitory role of NIM811 treatment on wound-healing rate (Supplemental Figure 3) as well as on reepithelialization after 1 week of treatment (Figure 3, B and C). Moreover, the amount and maturity of the granulation tissue decreased with NIM811 application (Figure 3, D, E, and H). Wound healing, reepithelialization, and granulation all returned to normal after 4 weeks in both vehicle- and NIM811-treated conditions (Figure 3, F, G, and I), suggestive of a role of CyD in early wound healing. Previous studies have shown that efficient granulation and healing are dependent on collagen deposition at the wound site (2). To test if CyD inhibition by NIM811 affected collagen deposition, we employed Masson’s trichrome and Sirius red staining on paraffin-embedded sections. Wounds subjected to NIM811 presented diminished collagen deposition (Figure 3, J and K). Taken together, these results indicate that CyD is enriched in the wound niche and supports both keratinocyte migration and collagen deposition, which collectively contribute to an efficient wound closure.

### TEM shows reduction of collagen gene expression and fibril size in Ppif-KO mouse skin.

**Figure 4:**
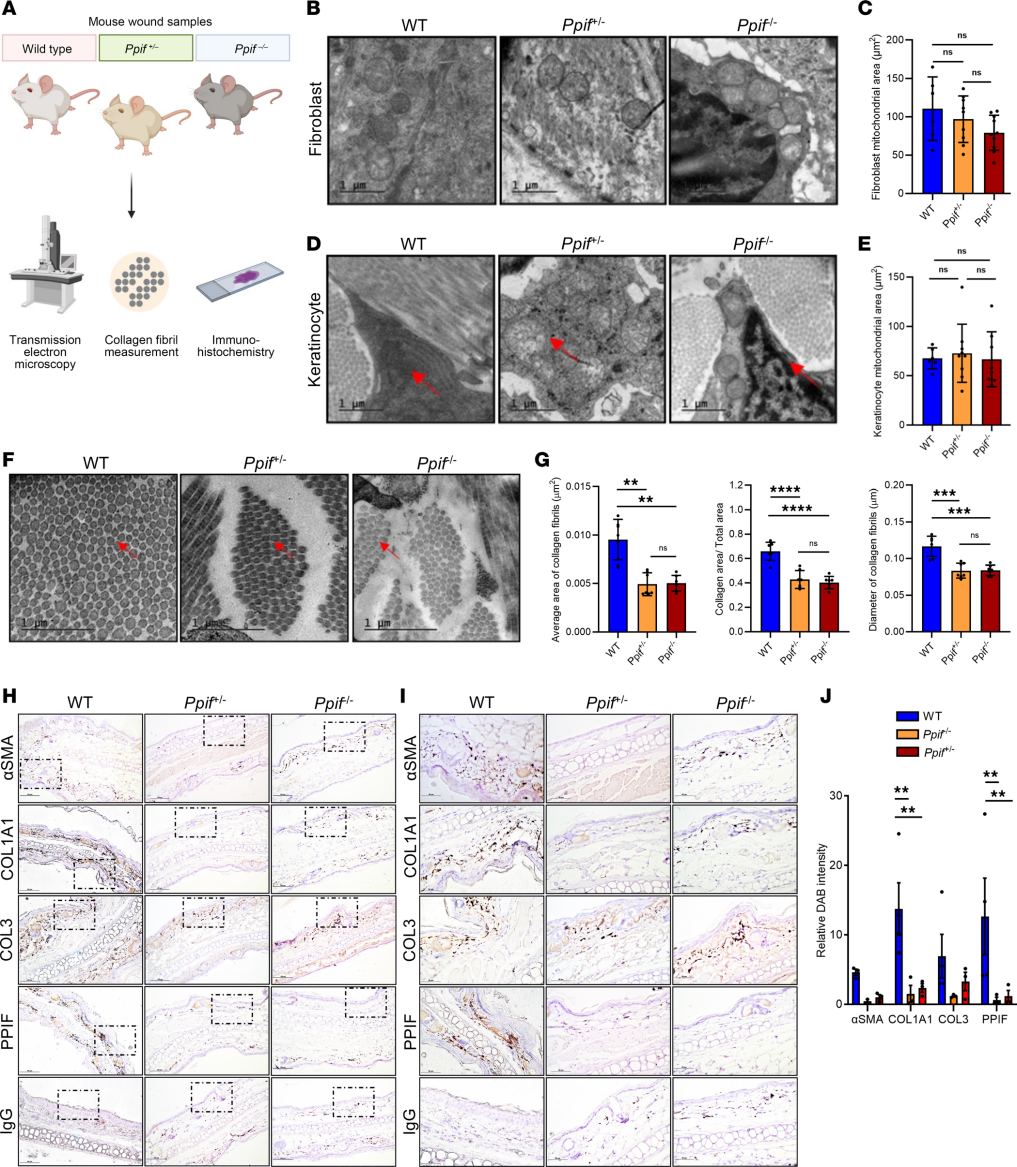
Skin collagen protein expression and fibrils are reduced in *Ppif*-KO mice while mitochondria appear normal. (**A**) Cartoon depicting the experimental setup. (**B**–**E**) Representative TEM micrographs of mitochondrial morphology (arrows) in keratinocytes (**B**) and fibroblasts (**D**), and quantification (mean ± SD) of mitochondrial area (**C**–**E**). *n* (number of mouse skin samples) = 7. Arrows indicate mitochondria. (**F** and **G**) Representative micrographs of collagen fibril morphology and density (arrows) as well as quantification (mean ± SEM) in fibroblasts. Note the reduction in fibril size and density. *n* = 7. (**H**–**J**) IHC images (**H**), zooms (**I**), and quantification (**J**) (mean ± SD) of skin sections stained with the main collagen types 1/3 and upstream collagen signaling marker α-SMA, CyD, and IgG. *n* = 3 mice per condition with 3 different visual fields. Scale bars: 1 μm (TEM); 100 μm; and 50 μm (IHC). Two-way ANOVA. *n* = 3. ****P < 0.0001; ***P < 0.001; **P < 0.01.

The results of the human explant wound healing assay and porcine model suggest a role of CyD in collagen fibrillogenesis, possibly independent of wound healing. To study this, we performed transmission electron microscopy (TEM) and IHC on skin samples from WT, *Ppif^–/+^*, and *Ppif^–/–^* mice (Figure 4A). Since CyD is a mitochondrial protein, we first measured mitochondrial area, as a mitochondrial mass parameter, in both keratinocytes and fibroblasts, but we found no significant differences between the mice (Figure 4, B–E). Moreover, there were no apparent changes in mitochondrial morphology (Figure 4, B–E). As no mitochondrial aberrations were found, we then performed in vitro respirometry experiments and subsequently found no inhibition of mitochondrial respiration with *PPIF* knockdown (KD) or overexpression (OE) (Supplemental Figure 4 and Supplemental Figure 5). We then measured both the area and diameter of collagen fibrils, and we found that both were reduced in *Ppif^–/+^* and *Ppif^–/–^* mice when compared with WT (Figure 4, F and G). While, in some images, collagen fibrils appeared less contiguous, this was not consistent and, therefore, not quantified.

Furthermore, we performed IHC in order to assess collagen deposition and myofibroblast differentiation, as well as to verify a reduction of CyD at the protein level (Figure 4, H and I). Here, we observed a significant reduction in collagen 1 staining in *Ppif^–/+^* and *Ppif^–/–^* mice (Figure 4, H–J). α-SMA, a common marker of myofibroblast differentiation, as well as CyD, were both reduced in *P*pif*^–/+^* and *Ppif^–/–^* skin samples. Altogether, these results suggest a role for CyD in collagen regulation not only in stressed conditions such as wound healing but also in normal skin, likely independent of mitochondrial function.

### CyD enhances keratinocyte migration, and transcriptomic analysis of PPIF KD predicts a key role for ECM formation.

**Figure 5:**
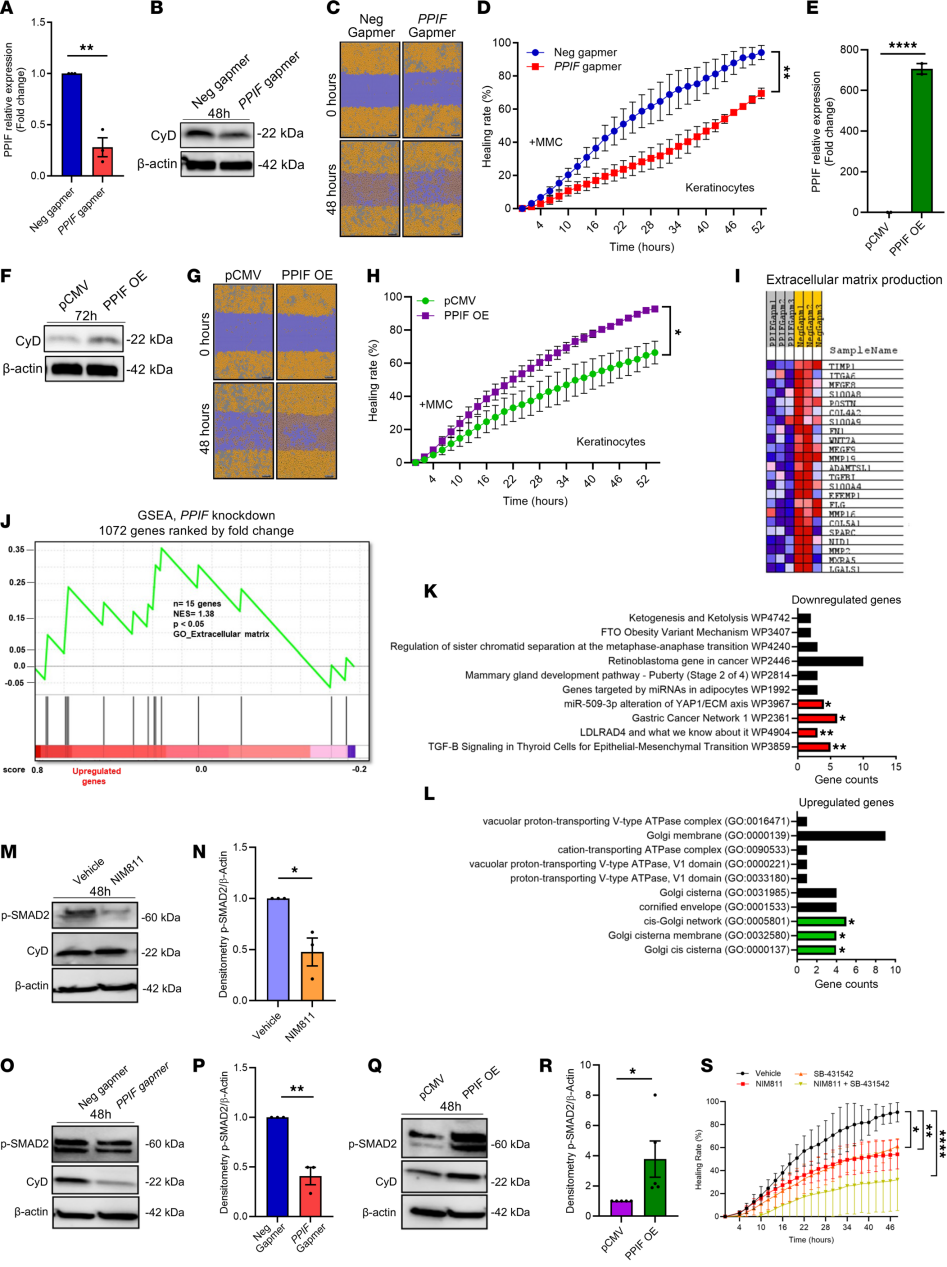
Cyclophilin D promotes keratinocyte migration and affects expression of extracellular matrix genes. (**A** and **B**) qPCR (mean ± SEM) and immunoblot verification of gapmer-mediated CyD KD compared with negative gapmer in keratinocytes. (**C** and **D**) Scratch cell migration assay in keratinocytes with gapmer-mediated *PPIF* KD in the presence of mitomycin C. *n* = 3; 2-tailed, unpaired *t* test. (**E** and **F**) qPCR (mean ± SEM) and immunoblot verification of vector-mediated *PPIF* OE compared with pCMV in keratinocytes. Two-tailed, unpaired *t* test. (**G**–**H**) Scratch cell migration assay in keratinocytes with *PPIF* OE in the presence of mitomycin C. One-way ANOVA. (**I**) Heatmap illustration of microarray analysis for differentially expressed genes after *PPIF* KD (fold change < 1.2 or > 1.2, with *P* < 0.05). Data were hierarchically clustered. Blue, lower; red, higher. *n* = 3 biological replicates. (**J**) GSEA heatmap and enrichment score showing a change in extracellular matrix–maintaining genes. (**K** and **L**) Molecular pathways identified by Enrichr software to understand the most important mechanism affecting the keratinocyte migration. (**M**–**R**) Immunoblot and quantification (mean ± SEM) of phosphorylated SMAD2 in vehicle and NIM811-treated (**M** and **N**), negative and *PPIF* gapmer (**O** and **P**), and pCMV and PPIF OE keratinocytes (**Q** and **R**). Two-tailed, unpaired *t* test. (**S**) Scratch cell migration assay in keratinocytes with NIM811 and SB-431542 treatment in the presence of mitomycin C. ****P < 0.0001; **P < 0.01; *P < 0.05.

To substantiate our human and porcine data, which suggest a decrease in wound healing with NIM811 treatment, and to examine the effect of different CyD levels on human primary keratinocytes, we performed either *PPIF* gapmer-mediated KD or OE by a pCMV/*PPIF* plasmid, followed by scratch assay. qPCR and immunoblotting confirmed KD and OE (Figure 5, A, B, E, and F) with no effect on cell viability or superoxide production (Supplemental Figure 6, A and B). Supporting a functional effect of CyD, calcein-cobalt chloride quenching assay employed for measuring mPTP activity showed a decrease in mPTP activity with *PPIF* KD and an increase in mPTP activity with OE (Supplemental Figure 6, C–E). Scratch assay also showed delayed wound healing with *PPIF* KD, while OE accelerated wound healing (Figure 5, C, D, G, and H).

To unravel potential noncanonical mechanisms for CyD in keratinocytes, we performed a transcriptomic analysis following *PPIF* KD. Microarray analysis showed 1,552 differentially expressed genes (*P* < 0.05) (FC ≥ 1.2), with a large majority of these genes being upregulated (Figure 5I). Gene set enrichment analysis (GSEA) demonstrated that a large panel of ECM-related genes were decreased with *PPIF* KD (Figure 5J). Consistent with the microarray gene expression data and GSEA, EnrichR analysis showed an increase in *TGFβ1* expression, signifying the importance of PPIF in the epithelial-mesenchymal interaction (Figure 5, K and L). qPCR expression analysis also demonstrated reduced expression of *SMAD2*, which mediates TGF-β signaling, in both *PPIF* KD and NIM811-treated keratinocytes, suggesting that CyD mediates keratinocyte migration through the TGF-β signaling pathway (Supplemental Figure 7, A–C). Consistent with this, *PPIF* KD and NIM811 treatment inhibited TGF-β–induced phosphorylation of SMAD2 as shown by immunoblotting (Figure 5, M–P), while *PPIF* OE displayed the opposite (Figure 5, Q and R). Scratch assay using SB-431542, a small molecule inhibitor of TGF-β, increased phosphorylation of SMAD2 and induced the inhibition of keratinocyte migration (Figure 5S). Wound healing subsequently increased when used in combination with the CyD inhibitor NIM811, further strengthening the possibility that CyD works through the TGF-β signaling pathway in keratinocytes (Supplemental Figure 5).

### CyD promotes fibroblast proliferation but not migration, and microarray data reveal a vital role in collagen formation.

**Figure 6:**
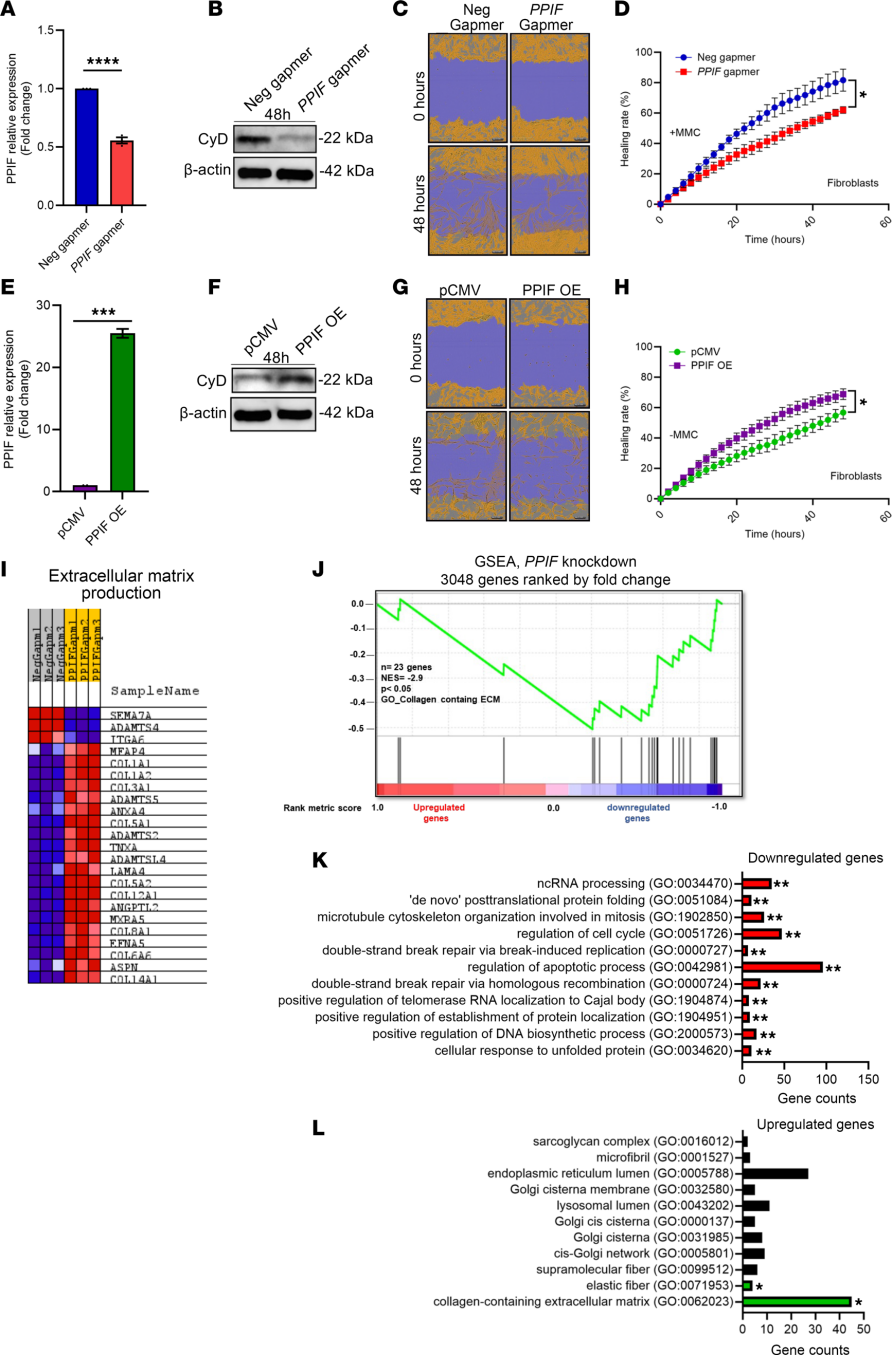
Cyclophilin D promotes fibroblast proliferation but not migration. (**A** and **B**) qPCR (mean ± SEM) and immunoblot verification of gapmer-mediated *PPIF* KD compared with negative gapmer in fibroblasts. Two-tailed, unpaired *t* test. (**C** and **D**) Scratch cell migration assay in fibroblasts with gapmer-mediated *PPIF* KD in the presence of mitomycin C. *n* = 3 biological replicates. (**E** and **F**) qPCR (mean ± SEM) and immunoblot verification of vector-mediated *PPIF* OE compared with pCMV in fibroblasts. (**G** and **H**) Scratch cell migration assay in fibroblasts with *PPIF* OE in the absence of mitomycin C. (**I**) Heatmap illustration of microarray analysis for differentially expressed genes after *PPIF* KD (fold change < 1.2 or > 1.2, with *P* < 0.05). Data were hierarchically clustered. Blue, lower; red, higher. *n* = 3 biological replicates. (**J**) GSEA heatmap and enrichment score showing a change in extracellular matrix–maintaining genes. (**K** and **L**) Molecular pathways identified by Enrichr software to understand the most important mechanism affecting the fibroblasts proliferation and extracellular matrix gene regulation. ****P < 0.0001; ***P < 0.001; **P < 0.01; *P < 0.05.

To complement the in vitro keratinocyte experiments, we performed similar experiments on fibroblasts with *PPIF* KD or OE (Figure 6, A–H) without observing an effect on either cell viability or superoxide production (Supplemental Figure 8, A and B). Contrary to that seen in keratinocytes, scratch assay experiments with fibroblasts did not show any change in healing rate in the presence of the proliferation inhibitor mitomycin C (MMC) (Supplemental Figure 8, C–F). Transient incubation with MMC for 2 hours inhibited the proliferation rate while maintaining the migration ability (30). However, in accordance with the human and porcine data, in the absence of MMC, we observed a significant decrease in proliferation with *PPIF* KD (Figure 6, C and D), as well as an increase in proliferation with *PPIF* OE (Figure 6, G and H). Similarly to keratinocytes, results from the calcein-cobalt chloride–quenching assay demonstrate a decrease in mPTP activity with *PPIF* KD and an increase in mPTP activity with OE in fibroblasts (Supplemental Figure 8, G–I).

To examine what cellular pathways were affected by *PPIF* KD, RNA from *PPIF* KD cells was used for microarray and qPCR analysis, where a total of 3,048 differentially expressed genes were detected (Figure 6I). Importantly, through GSEA, we observed an upregulation in the expression of collagen-related genes (Figure 6J). Furthermore, EnrichR analysis also demonstrated that the highest alterations were in ECM-related pathways, further suggesting a role of CyD in collagen-mediated pathway regulation (Figure 6, K and L). However, EnrichR analysis showed a decrease in expression of cell cycle and protein folding genes (Figure 6K).

### CyD regulates a gene network of ECM and collagen 1 both at production and secretion level in human dermal fibroblasts.

**Figure 7:**
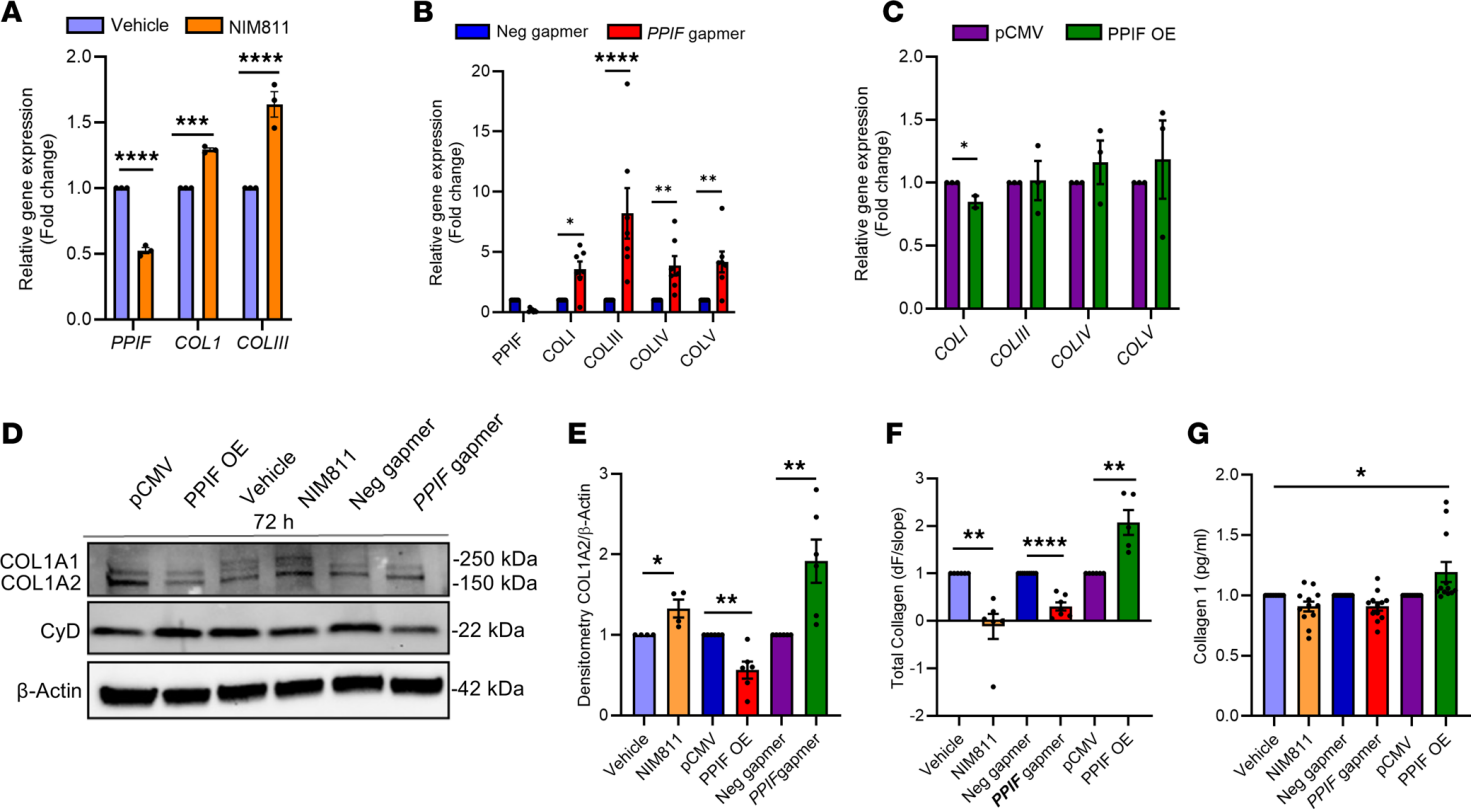
Cyclophilin D levels affect collagen synthesis and secretion in human dermal fibroblasts. (**A**–**C**) qPCR (mean ± SEM) of collagen type 1, 3, 4, and 5 in vehicle and NIM811-treated (**A**), *PPIF* KD (**B**), and PPIF OE in fibroblasts (**C**). Two-tailed, unpaired *t* test. (**D** and **E**) Immunoblot and densitometric quantification (mean ± SEM) of collagen 1 in fibroblast lysates. Two-tailed, unpaired *t* test. Note that the *PPIF* expression is increased in *PPIF* gapmer and NIM811 as compared with negative gapmer and vehicle, respectively; however, *PPIF* expression is diminished after *PPIF* OE as compared with vector alone. (**F**) Ratio (mean ± SEM) of total collagen in media (extracellular, secreted) to lysate (intracellular) in fibroblasts using glycine-based enzymatic fluorescence assay. (**G**) Collagen 1 secretion to media by fibroblasts measured by specific ELISA (mean ± SEM). *n* = 3 biological replicates. Two-way ANOVA. ****P < 0.0001; ***P < 0.001; **P < 0.01; *P < 0.05.

Collagen synthesis occurs in several locations, including in the endoplasmic reticulum (ER), Golgi body, and lysosomes, before its secretion and deposition in the ECM (31–34). To study how *PPIF* KD, OE, or pharmacological inhibition affected this, we performed several experiments on human dermal fibroblasts. Firstly, gene expression of several collagens was elevated with both *PPIF* KD or NIM811 treatment but, conversely, was unchanged with *PPIF* OE (Figure 7, A–C). Moreover, immunoblot analysis of cell lysate showed an increase in the protein expression of collagen 1 following *PPIF* KD and NIM811 treatment, as well as a decrease in its expression with *PPIF* OE (Figure 7, D and E). By contrast, there was no significant change in collagen 3 protein expression with either *PPIF* KD or OE (Supplemental Figure 9, A and B).

Secondly, we then examined collagen secretion by comparing collagen concentration in the culture media versus in cell lysate, using a glycine-based collagen assay (35). Here, we observed a decreased secretion of overall collagen with both *PPIF* KD or NIM811 treatment, while *PPIF* OE increased collagen secretion (Figure 7F). Thirdly, we measured collagen 1 concentration in the cell media using collagen 1–specific ELISA, which revealed a significant increase in collagen 1 secretion after *PPIF* OE (Figure 7G). However, there was no change in collagen 3 secretion, suggesting a specific role of CyD in secretion of collagen 1 (Supplemental Figure 9C). Finally, to rule out the possibility that collagen secretion was affected by mitochondrial dysfunction, we performed Seahorse mitochondrial stress test assessment on both fibroblasts as well as keratinocytes. This analysis showed no change in respiration with either *PPIF* OE or KD (Supplemental Figures 4 and 5), suggesting that mechanisms other than mitochondrial dysfunction are causing the change in collagen 1 secretion by CyD.

### CyD inhibition leads to collagen accumulation in the ER and inhibits its secretion outside the cell.

**Figure 8:**
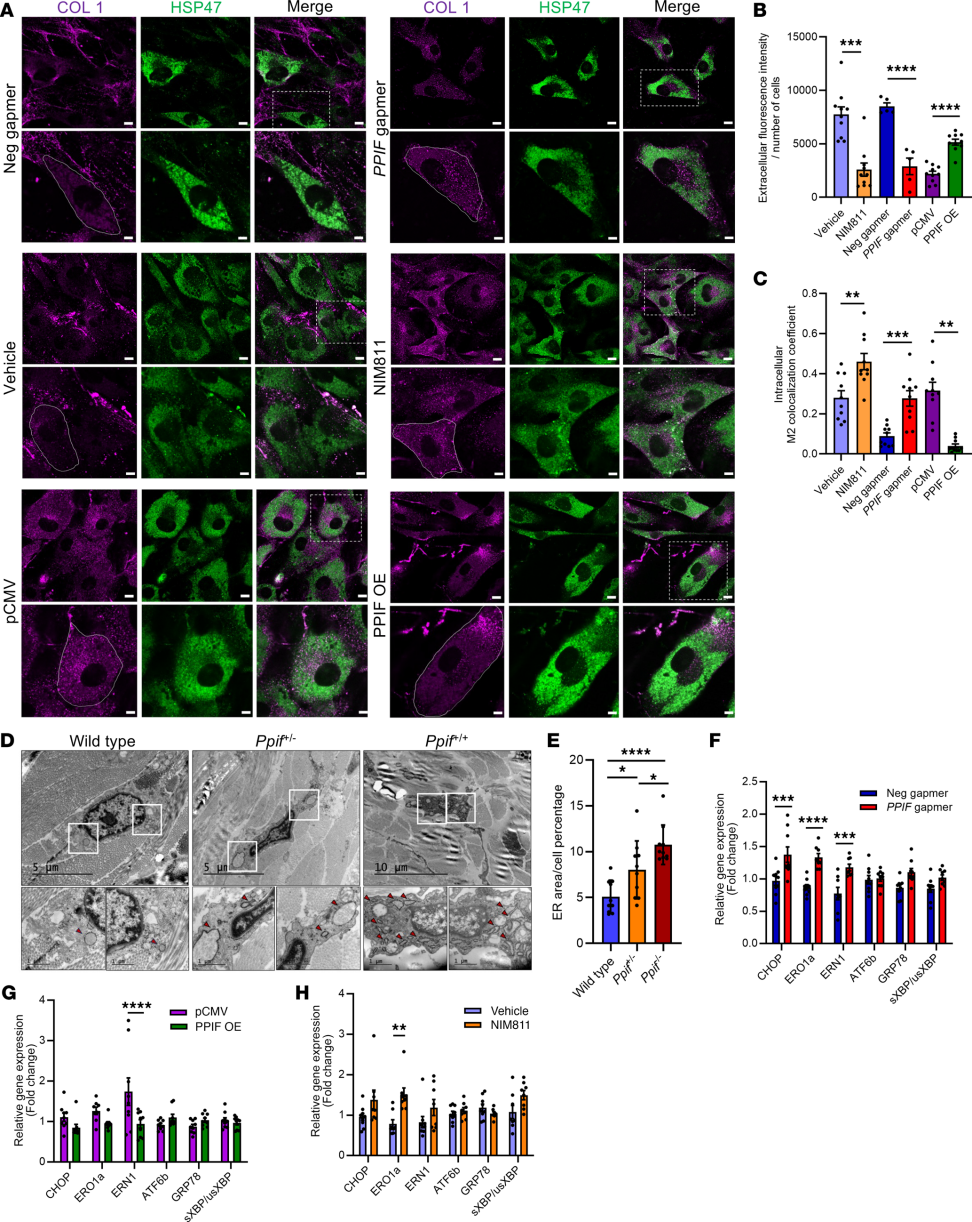
Cyclophilin D inhibition causes ER aggregation of collagen 1 while its OE increases collagen 1 secretion in human dermal fibroblasts. (**A**) Assessment of intra- and extracellular collagen 1 by confocal immunofluorescence imaging. The white lines depict the outlines of fibroblast cells. Zooms are depicted underneath images. Scale bar: 10 μm (full), 5 μm (zoom). (**B**) Quantification (mean ± SEM) of extracellular collagen 1 fluorescence intensity. (**C**) Colocalization coefficient analysis (mean ± SEM) of collagen 1 with ER marker HSP47 M2. Two-way ANOVA. (**D**) Representative TEM images of fibroblasts in the skin of WT, *Ppif*^+/–^, and *Ppif^–/–^* mice. Scale bar: 5 μM (left and middle panels) and 10 μM (right panel) (full size); 1 μM (zoom). Red arrows depict ER expansion. (**E**) Quantification (mean ± SEM) of ER size. Two-way ANOVA. (**F**–**H**) qPCR (mean ± SEM) of ER stress genes in gapmer-mediated *PPIF KD* compared with negative gapmer (**F**), PPIF OE compared with pCMV (**G**), and NIM811- compared with vehicle-treated fibroblasts (**H**). *n* = 3 biological replicates for all experiments. Two-tailed, unpaired *t* tests. ****P < 0.0001; ***P < 0.001; **P < 0.01; *P < 0.05.

To further investigate the collagen synthesis and secretion at the intracellular as well as extracellular level, we performed immunofluorescence experiments on fibroblasts using antibody markers for collagen 1 and the ER marker HSP47 (Figure 8A). Here, extracellular collagen decreased in response to both *PPIF* KD and NIM811-induced inhibition following 7 days of treatment, while *PPIF* OE led to a significant increase in extracellular collagen 1 (Figure 8, B and C). Correspondingly, there was a decrease in intracellular collagen with *PPIF* OE and an increase in intracellular collagen with *PPIF* KD (Figure 8, B and C). Downregulation of *PPIF*, thus, led to disrupted intracellular collagen 1 trafficking and accumulation of collagen in the ER, while in contrast, enhanced trafficking and secretion followed *PPIF* OE (Figure 8C). To validate the accumulation of collagen 1 in the ER, we performed an ER enrichment experiment, followed by ELISA for collagen 1. Collagen 1 accumulation was increased in the ER in NIM811-treated cells, further suggesting that CyD inhibition leads to collagen accumulation in the ER (Supplemental Figure 10, A and B). TEM analysis in *Ppif*-KO mice fibroblasts demonstrated an increase in ER expansion (Figure 8, D and E), while qPCR experiments showed an increase in the gene expression of unfolded protein response (UPR) genes with *PPIF* KD (Figure 8, F–H). Additionally, confocal microscopy with *Ppif* downregulation showed an increase in cisternal ER size, as well as a decrease in tubular ER size in cultured fibroblasts (Supplemental Figure 10, C and D).

## Discussion

This work presents a role for the mitochondrial matrix protein CyD, upregulated in human wound biopsies, in 2 vital processes involved in wound healing — reepithelialization and granulation tissue formation. In keratinocytes, CyD promoted reepithelialization possibly by activating TGF-β receptor–mediated signaling through SMAD2. In fibroblasts, CyD had a role in fibroblast proliferation, ECM gene regulation, and collagen secretion, with its inhibition leading to reduced collagen deposition and intracellular ER accumulation and with its OE leading to elevated collagen deposition. This was substantiated by 2 in vivo models — in CyD-deficient mice, where extracellular collagen fibrils were reduced, as well as in NIM811-treated pig wounds, where there was a reduction in granulation tissue formation and subsequently delayed wound closure.

### Canonical and noncanonical roles of CyD.

CyD is a mitochondrial peptidyl-prolyl *cis-trans* isomerase, is a member of the larger cyclophilin family, and is foremost recognized for its role in regulating the mPTP pore (14, 36). CyD activity is regulated by posttranslational modifications such as acetylation, phosphorylation, and nitrosylation (37). Although the exact mechanism is still unknown, CyD stimulates mPTP opening (14), which classically leads to cytochrome C release into the cytoplasm and activation of the intrinsic apoptotic pathway (38). Recent literature has suggested new noncanonical functions of CyD independent of mPTP opening (37). For example, CyD interacts with B cell lymphoma 2 (BCL2) to mediate cytochrome C release from mitochondria, thereby initiating apoptosis independently of mPTP opening (39). Of note, CyD inhibition or downregulation has also been reported to enhance neuronal, cardiomyocyte, and osteoblast differentiation (40–42).

Our study identifies previously unknown noncanonical roles for CyD in wound healing and collagen secretion during healthy wound healing. Furthermore, our results are unlikely to be explained by indirect effects of CyD on mitochondrial bioenergetic function, as CyD reduction nor OE altered mitochondrial respiration in either keratinocytes or fibroblasts (Supplemental Figures 4 and 5), while both mitochondrial mass and morphology appeared unchanged in *Ppif*-KO mouse skin (Figure 4, B–E). This differs from other studies that reported that *Ppif* KO shifts metabolism in skeletal muscle, bone, liver, heart, and kidney toward glycolysis and an increase in fatty acid oxidation (43). This is perhaps reflective of different metabolic regulation in the skin. Thus, CyD may have specific functions in the skin, and at least some are likely not mPTP dependent.

### CyD and wound healing.

Reepithelialization of wounded tissue, an early-phase process in wound healing, is primarily performed by keratinocyte proliferation and migration from wound edges (44). Little is known about the link between CyD and reepithelialization or its general role in human skin physiology. In mice, *Ppif* KO was associated with acceleration of skin wound healing, possibly through increased angiogenesis (45); however, opposite results were reported in another *Ppif* KO study, which showed healing decelerated associated with platelet dysfunction (46). Furthermore, in rats, the unspecific CyD inhibitor cyclosporine A caused delayed wound healing (47, 48). To the best of our knowledge, there are no data on the role of CyD in wound healing in humans or animals with similar skin physiology to humans, such as pigs. While not being a wound model, ultraviolet B–induced (UVB-induced) human keratinocyte cell death was shown to be dependent on CyD, in that KD cells were protected against cell death (38). No studies have reported how CyD may regulate wound healing independently of cell migration and proliferation in the skin; however, in cancer cell lines, CyD inhibition was associated with increased cell proliferation and enhanced cell migration, possibly by elevated STAT3 proinflammatory signaling (49).

In this study, we observed an increase in CyD expression in both acute and venous ulcer samples, both at the gene and protein level, as well as matching alterations in mPTP opening and CyD expression. This was corroborated in the reanalysis of a published data set derived from similar samples, where pressure ulcer samples displayed a decrease in *PPIF* gene expression (50). To examine the role of CyD in wound healing, we performed a series of in vitro, ex vivo, and in vivo experiments — undertaking a comprehensive assessment on this topic. Genetic and pharmacological CyD inhibition showed a clear inhibition in epidermal reepithelialization and keratinocyte migration, possibly by inducing ECM-related genes including *TGFβ1* and its downstream target *SMAD2*. Indeed, activation of TGF-β signaling was previously shown to promote keratinocyte migration and reepithelialization during acute wound healing and in tissue regeneration (51), and it was shown to promote other wound healing features such as angiogenesis (51). In contrast, in CyD downregulated and NIM811-treated fibroblasts, we propose decreased DNA biosynthesis and altered cell cycle kinetics as a mechanism for impaired proliferation (52), while impaired collagen secretion underpins reduced granulation as a result of impaired collagen processing in the ER, as discussed in detail below. Describing and deciphering the role of CyD in wound healing is important clinically; many patients are still treated with cyclosporine A as well as for future development of potentially novel CyD-interacting molecules (53).

### CyD and collagen.

Fibroblast proliferation and ECM deposition are interdependent processes activated following wounding and are both key determinates of tissue repair (2, 54, 55). Collagen 1, an evolutionary ancient protein and one of the most common proteins overall in higher organisms (56), is the prime constituent of ECM in skin, tendon, and other tissues, and its expression and deposition is regulated at both the translational and posttranslational levels by different stimuli and pathways such as TGF-β signaling (57–59). However, critical questions concerning the collagen secretion pathway, including the exact mechanism of collagen folding, remains to be investigated, while data on CyD and collagen remain lacking. Recently, a role of cyclophilins in amino acid modification of collagen and its crosslinking was observed in skin, where deletion of the ER-resident cyclophilin B reduced collagen glycosylation, hydroxylation, and fibrillogenesis (19, 60–62). Previous literature has also indicated a role for cyclophilins in collagen folding, whereby cyclophilin A and B activity reduced SMAD2 phosphorylation and lysine hydroxylation in collagens, thus impeding collagen fibrillogenesis and structural organization (20, 63). Somewhat unexpectedly, in our study, we found that inhibition of CyD disrupted collagen 1 deposition, structure, and fibrillogenesis, both through in vivo pig and *Ppif*-KO mice model experiments; ex vivo in human explant wounds; and in vitro experiments using primary human fibroblasts. Collectively, these results suggest that downregulation of CyD contributes to an abnormal regulation of the granulation process, especially with regard to collagen secretion; thus, it may interfere with wound healing. This data extend and explain a previous unspecific observation on how the nonspecific CyD inhibitor cyclosporine A increased expression of collagen 1 mRNA (64), and they suggest that CyD may be of interest therapeutically. Mechanistically, although CyD inhibition increased collagen gene expression, secretion was impaired, likely due to ER accumulation of procollagen. This increase in collagen gene expression may be due to reduced transcriptional feedback, perhaps as a result of reduced collagen binding to cell-surface integrin receptors (65). Additionally, the decrease in collagen secretion with *PPIF* downregulation may also be due to an increase in the UPR, as suggested by an increase in ER stress gene expression. Elevated UPR activation could also lead to the ER expansion, as shown by TEM and confocal microscopy. The demonstrated role of CyD in collagen synthesis and secretion identified herein adds to the understanding of collagen synthesis trafficking in general as well as wound healing specifically.

### Fibrotic disease.

After tissue injury in healthy organs, fibroblasts produce and secrete more collagen in order to maintain tissue integrity (66). However, when fibroblasts secrete and deposit excessive collagen, it disrupts the tissue equilibrium and leads to fibrosis, which can affect several organs (67). Therefore, there is a great clinical need for developing novel collagen-reducing compounds for the treatment of fibrotic disease, since available treatments are scarce and often inefficient (68, 69). Here, we show that both pharmacological and genetic inhibition of CyD limits collagen secretion and deposition and, therefore, may have pathogenic implications for several fibrotic conditions of the skin. Regarding other organs, antifibrotic effects of NIM811 and the cyclophilin A inhibitor MM284 were observed in cardiac fibrosis models (70). However, in this particular study, the collagen deposition mechanism was unclear. Furthermore, NIM811 treatment was also shown to attenuate liver fibrosis (71), while CyD was shown to contribute to kidney fibrosis by inducing proinflammatory and profibrotic factors in obstructive neuropathy (72). Thus, ours and others data suggest that CyD may be a therapeutic target in fibrotic diseases in which collagen reduction is the goal. Moreover, the finding that CyD OE increases collagen deposition suggests that it may be a therapeutic target in conditions with too little collagen such as skin aging or glucocorticosteroid-induced dermal atrophy.

### Conclusions.

CyD is important for human skin wound healing and, in particular, appears to affect granulation tissue formation, collagen secretion, and deposition. Future studies should examine what effect increased CyD activity has on wound healing as well as collagen deposition in vivo.

## Methods

Detailed protocols for methods and materials carried out for human sample collection, ex vivo treatment on pigs, TEM, cell culture, NanoString analysis, qPCR, Western blotting, microarray, respirometry, cell viability, immunofluorescence, confocal microscopy, glycine-based collagen assay, ELISA for collagen 1 and 3, and IHC are provided below and in Supplemental Methods. Antibodies and oligos used in this study are listed in Table 1, Table 2, and Table 3.

### Sex as a biological variable.

Skin biopsies were taken from both male and female donors, although sex was not considered as a biological variable.

### Human skin and wound biopsies.

Healthy volunteers (>60 years of age) were enrolled at Karolinska University Hospital Dermatology Clinic, Stockholm, Sweden. Full-thickness skin wounds were made with a 4 mm biopsy punch in 2 adjacent spots 2 cm apart on the distal lower leg of the healthy volunteers — the excised skin was then used as controls for the following wounds. On day 1 and day 7 after injury, the wound edge area was excised with a 6 mm biopsy punch from one of the existing wounds. Similar wound edge biopsies were obtained on 1 occasion from patients (> 60 years of age) with chronic C6 venous ulcers for > 3 months (73). All samples were snap frozen in liquid nitrogen.

### NanoString analysis.

Skin biopsies were homogenized using TissueLyser LT (Qiagen) prior to RNA extraction. Total RNA was extracted from human tissues using the miRNeasy Mini kit (Qiagen). We performed a custom designed nCounter assay (NanoString Technologies) for determining the mRNA expression of genes involved in various metabolic pathways. nCounter gene expression was conducted in the samples from harvested wound punch biopsies from healthy volunteers at day 0, day 1, and day 7 following the wound induction as well as from subjects with chronic venous leg ulcers. We used Molecular Signatures Database (MSigDB) to create a list of genes involved specifically in apoptosis, necrosis, and CyD interaction. In addition to the 40 genes listed in the heatmap, there were 6 housekeeping genes, 6 positive control genes, and 8 negative control genes. RNA extracted from skin biopsies from healthy volunteers and patients with chronic venous ulcers were hybridized with reporter-capture probe pairs according to the manufacturer’s instructions. Hybridized probes were then purified and immobilized in the nCounter cartridge (NCT-120, NanoString Technologies). Data collection was done using the nCounter Digital Analyzer (NanoString Technologies) in order to quantify target RNA molecules present in each sample. Quantification of read-count data including differential expression and pathway analysis was performed using the NanoString nCounter nSolver 4.0 Advanced Analysis Module (MAN-10030-03, NanoString Technologies) as per the instructions for NanoString gene expression data analysis. Counts of the probes were normalized to reference genes obtained from geNorm algorithm. A 1.2-fold change and Benjamini-Yekutieli (BY) adjusted *P* value of < 0.05 was used to create the heat map of differentially expressed genes (DEGs).

### RNA in situ hybridization.

Chromogenic RNAScope was performed on formalin-fixed parrafin-embedded (FFPE) day 0, day 1, and day 7 wound sections (10 μm) from 5 patients using a standardized protocol (detailed in Supplemental Methods). Sections were then imaged at both ×10 and ×40 magnification, and the frequency and area of positive *PPIF* red dots normalized to the area of the section was quantified in both the dermis and epidermis of each section using ImageJ 1.53a software (NIH).

### Immunofluorescence.

For immunofluorescence of PPIF in human wound day-0, day-1, and day-7 skin, FFPE sections (10 μm) from 5 separate healthy control patients were deparaffinized and rehydrated as standard before standardized immunofluorescence staining (detailed in Supplemental Methods). After imaging with a Zeiss LSM 880 confocal at ×40 magnification, the fluorescence intensity of PPIF was quantified on ImageJ 1.53a for both the epidermis and dermis in each day 0, day 1, and day 7 section from each respective patient after background correction using the fluorescence intensity from a no primary control and then subsequently normalized to the fluorescence intensity for day 0 for each respective patient.

For immunofluorescence of intra- and extracellular collagen 1 in fibroblasts, after respective treatment or transfection, fibroblasts were washed twice with PBS and fixed with 4% paraformaldehyde for 15 minutes. Cells were again washed with PBS and permeabilized with 0.1% Triton X-100 in PBS for 15 minutes followed by another wash cycle. Permeabilized cells were blocked with 3% normal goat serum (Thermo Fisher Scientific) for 1 hour at room temperature (RT) and incubated with primary antibody at 4°C overnight. The next day, cells were washed with PBS 3 times and incubated with secondary antibody (1 hour at RT) and DAPI (10 minutes at RT). Finally, cells were washed and imaged with a Zeiss LSM 880 confocal microscope at ×20 and ×63 magnifications. Images were analyzed in ImageJ 1.53a and Zeiss Zen 3.0 software.

### Human skin explant ex vivo model.

Normal full-thickness human skin samples were obtained from abdominal reduction surgeries of healthy young donors (Nordiska Kliniken) and used for ex vivo wound healing as previously described (74) (Supplemental Methods). Differences in the area of initial wound and the remaining open wound were expressed as the percentage of wound closure at each time point.

### Mice.

C57BL/6J mice were obtained from The Jackson Laboratory and fed a chow diet (NIH-31M diet) from Taconic Biosciences. Whole-body *Ppif*-KO and littermate WT mice were produced from heterozygous breeding pairs derived from a male KO mouse (stock no. 009071, B6;129-Ppiftm1Jmol/J; The Jackson Laboratory) (75) and a female WT control mouse (stock no. 101045, B6129SF2/J; The Jackson Laboratory). Mice were housed at the UCLA Animal Resources Facility in a 12-hour/12-hour light-dark cycle and maintained at 20°C–22.2°C. Animals were provided water and food ad libitum.

### qPCR for human explants.

Skin biopsies were treated with vehicle or NIM811 (5 μM) for 1 and 7 days. At these days, treated skin samples were homogenized using TissueLyser LT (Qiagen) for 10 minutes prior to RNA extraction. Total RNA was extracted from human skin tissues using Trizol reagent (Thermo Fisher Scientific). After monitoring the quality of sample, reverse transcription was performed using the RevertAid First Strand cDNA Synthesis Kit (Thermo Fisher Scientific). Gene expression was quantified by SYBR Green expression assay (Thermo Fisher Scientific) and normalized with GAPDH. The primer information used in this experiment is listed in Table 1.

### Hydroxyproline-based collagen assays.

For assessing the collagen deposition in healthy and NIM811-treated human skin samples, we used hydroxyproline assay kit (Chondrex). Vehicle and NIM811-treated human skin biopsies were hydrolyzed in 6M HCl in a 100°C water bath for 4 hours in a 1:10 ratio of tissue weight to acid volume. In total, 100 μL supernatant was then incubated with 1× chloramine T solution provided within the kit for 20 minutes at RT. This mixture was then added to DMAB solution and incubated at 60°C for 30 minutes. Absorbance was measured at 530–560 nm in a spectrophotometer (SpectraMax iD3 plate reader), and hydroxyproline levels were calculated based on the standard curve plotted with the collagen standards provided in the kit.

### mPTP opening assay.

Calcein fluorescence was measured using the image-iT live mitochondria permeability transition pore kit (Thermo Fisher Scientific). In total, 30,000 cells were seeded in an 8-chamber plate (Ibidi). The next day, cells were loaded with calcein AM (1 μM), MitoSOX Red (500 nM) (Thermo Fisher Scientific) (both 30 minutes), and DAPI (15 minutes) with or without CoCl_2_ (1 mM) in HBSS (Sigma-Aldrich). Cells were then washed with HBSS, and images were taken with a Zeiss LSM 780 confocal microscope using a ×20 objective. Fluorescence intensity was analyzed on ImageJ 1.3a software.

### Porcine in vivo wound healing model.

The pig study was conducted in 1-year aged Göttingen minipigs (performed at MD Bioscsiences) where four 2 × 2 cm full-thickness skin wounds were inflicted on the back skin and 2 wounds were inflicted on the leg skin (Figure 3). Vehicle (ethanol) or NIM811 was applied topically every other day on the wounds until euthanasia. Wounds were harvested at both 1 and 4 weeks after induction, fixed in 10% formalin, and cut in the middle of the wounds for histology. The tissues were processed, embedded in paraffin, and sectioned for H&E staining. Wound closure was measured using the Silhouette star camera, and the wound area at each day was compared with the wound area of day 0 in order to calculate the percentage of area reduction. The main parameters used for healing were reepithelialization and granulation tissue deposition based on the progression of epithelial tongue and collagen deposition, respectively. In addition, Masson’s trichrome and Herovici staining were also performed on the histology slides for the measurement of collagen. The Herovici combination is a combination of picro methylene blue or aniline blue and picro acid fuchsin in proper proportions, where mature dense collagen appears red while newly formed collagen appears blue (76).

### TEM.

Skin samples were obtained from *Ppif*-KO mice with 4 mm punch biopsy and fixed in glutaraldehyde before TEM assessment of collagen fibrils and mitochondria. Briefly, TEM was performed after the fixation, dehydration, and embedding of WT, *Ppif^–/+^*, and *Ppif^–/–^* mice skin samples on ultrathin (60 nm) sections using a FEI Technai G2 transmission electron microscope at the Biovis imaging facility at Uppsala University, (Uppsala, Sweden).

### IHC.

IHC was performed on paraffin-embedded WT, *Ppif^–/+^*, and *Ppif^–/–^* mice skin sample sections using DAB-based IHC (Supplemental Methods). Protein expression levels were measured by QuPath software and then ImageJ 1.53a analysis of staining intensity divided by IgG staining intensity. The antibody information used in this experiment is listed in Table 2.

### Cell culture, transfection, and treatments.

Human epidermal keratinocytes (HEKa) were purchased from (Thermo Fisher Scientific, catalog C0055C) and cultured in EpiLife serum free keratinocyte growth media supplemented with human keratinocyte growth supplement (HKGS) (Thermo Fisher Scientific) and antibiotics (100 units/mL penicillin and 100 μg/mL streptomycin) (Thermo Fisher Scientific) in an incubator maintained at 37°C and 5% CO_2_. Primary human fibroblasts were isolated from healthy volunteers and cultured in DMEM glucose-free media (Thermo Fisher Scientific) supplemented with 10% FBS, 10 mM glucose, and antibiotics as above in an incubator maintained at 37°C and 5% CO_2_. *PPIF* KD was performed using 25 nM locked nucleic acid (LNA) long RNA GapmeR antisense oligonucleotides (Qiagen) for 48 hours by transfection with Lipofectamine 2000 (Thermo Fisher Scientific). PPIF OE was induced by *PPIF* human-tagged ORF clone (NM_005729) (Origene), with pCMV6 vector (Origene) acting as a nontargeting control. To study the inhibition of CyD activity and its effect on keratinocytes and fibroblasts, the specific pharmacological inhibitor NIM811 was used for 48 hours. The Gapmer information used in this experiment is listed in Table 3.

### Immunoblotting.

Immunoblotting was performed to verify the change in protein expression of CyD and other markers. Whole-cell lysate fractions were isolated from transfected and NIM811-treated cells (Supplemental Methods). The antibody information used is listed in Table 2. We first normalized the intensity of each band to the intensity of the β-actin of the same sample and then divided the values by the value of the day 0 sample of the same donor. To normalize the relative intensity of CW samples to day 0 controls, both the gels were run in parallel and intensity of each CW was divided by day 0 intensity of the sample on the same gel. β-Actin was used as a loading control; following appropriate stripping, the same membranes were reprobed for β-actin.

### Scratch assay.

Human dermal fibroblasts and keratinocytes were seeded into 96-well Incucyte plates (Sartorius) at a density of 20,000 keratinocytes or 17,000 fibroblasts per well. After reaching 90% confluency, cell monolayers were scratched using WoundMaker (Sartorius) and were washed twice with PBS to remove cell debris. Afterward, media were added to scratched cells. The plates were monitored for 48 hours in an Incucyte Live-Cell Analysis Systems (Sartorius), with imaging at 2-hour intervals. The migration area of cells was measured using the Incucyte Zoom 2018A and was plotted against time.

### Microarray and functional analysis.

Fibroblasts and HEKa were transfected with either negative or *PPIF* Gapmer for 72 hours and 48 hours, respectively, and were used for RNA extraction. RNA was extracted as stated previously, and RNA quality and quantity was analyzed with Nanodrop 1000. In total, 100 ng of the total RNA was used for cDNA preparation and qPCR analysis of *PPIF* gene expression. RNA from samples showing at least 4-fold *PPIF* KD were sent for expression profiling using Affymetrix cartridge at the core facility for bioinformatics and expression analysis at Karolinska Huddinge Department (Karolinska Institutet). Affymetrix standardized process including hybridization, fluidics processing, and scanning were used for gene expression. Genes showing at least a 1.2-fold change with *P* < 0.05 after *PPIF* KD were used for creating a list of differentially expressed genes. Theoretically, high FC cutoff can increase the false negative rate; therefore, a FC ≥ 1.2 cutoff for statistically significant changes in qPCR and microarray was used. Enrichr analysis (https://maayanlab.cloud/Enrichr/) (77) and GSEA (https://www.gsea-msigdb.org/gsea/index.jsp) (78) were performed in order to understand the functionally altered pathways after *PPIF* KD. A heatmap diagram was generated using the Multiple Experiment Viewer (MEV) software (https://sourceforge.net/projects/mev-tm4/) (79).

### ELISA.

For ELISA assessment, collagen 1 (Abcam, ab210966) and Collagen 3 (Cusabio, CSB-E13446h) kits were used. Ninety-six–well plates precoated with collagen antibodies were incubated with samples and standard controls as mentioned in the kit’s protocol. Plates were washed 3 times with PBST using an automatic ELISA microplate washer. Washing was followed by incubation with secondary antibody and addition of TMB-1 substrate component in the dark. To stop the reaction, stop reagent was added and plates were read at 450 nm in a SpectraMax iD3 plate reader. Each sample was measured in duplicate, and at least 3 biological repeats were conducted for each experiment.

### OCR and ECAR measurement.

Oxygen consumption rate (OCR) and extracellular acidification rate (ECAR) were measured by extracellular flux analyzer XF24e (Seahorse Bioscience) using mitostress and glycostress tests, respectively. Briefly, cells were seeded at 30,000 per well for fibroblasts and 40,000 per well for keratinocytes in XF24-well plates (Agilent Technologies) in 200 μL of respective cell culture media and incubated for 24 hours at 37°C and 5% CO_2_ prior to the experiment. For OCR measurement, cell media were replaced with 100 μL of XF assay medium (Aligent Technologies) supplemented with 2 mM glutamine (Sigma-Aldrich) and placed in an incubator without CO_2_ for 1 hour while the probe cartridge calibration was completed. Drugs were then added in the cartridge in the sequence as follows: port A: oligomycin (1 μM) (Sigma-Aldrich); port B: FCCP (0.5 μM) (Tocris); and port C: rotenone (2 μM) and antimycin A (1 μM) (both Merck). After completion of cartridge calibration, cell plate was added and basal OCR measured. Similarly, for ECAR measurement, cell medium was replaced with XF medium supplemented with 2 mM glutamine and placed in an incubator without CO_2_ for 1 hour while the probe cartridge calibration was completed. Basal ECAR was measured in the medium without the addition of glucose and glycolysis and was measured by injecting glucose (10 mM) in port A, oligomycin (1 μM) in port B, and 2-Deoxyglucose (2-DG) (50 mM) (Sigma-Aldrich) in port C. Finally, data were normalized to protein content.

### ER enrichment experiment.

ER Enrichment Kit (Invent Biotechnologies) was used to isolate ER from fibroblasts according to the protocol provided. Briefly, 1.8 × 10^7^ fibroblasts were used for vehicle and NIM811 treatment. After 48 hours of treatment, cells were spun down and buffer A was added. Then the cell suspension was transferred to a filter cartridge and centrifuged twice (16,000*g*, 30 seconds, 4°C). After centrifugation, supernatant was transferred to a fresh tube, and buffer B was added before centrifugation. The pellet was resuspended in cold buffer A and vortexed vigorously. Next, buffer C was added, followed by brief vortexing. Supernatant was then transferred to a fresh tube and buffer D added. After centrifugation (8,000*g*, 10 minutes, 4°C), the pellet was dissolved in PBS supplemented with protease inhibitors, and protein concentration was quantified. Finally, ER protein was used for conducting collagen 1 ELISA.

### Cellular viability.

The cell viability at various concentrations of all pharmacological metabolic drugs was determined using resazurin-based PrestoBlue assay. After the addition of Prestoblue (Thermo Fisher Scientific), cell absorbance at 560 nm was monitored using the SpectraMax iD3 plate reader, and the absorbance values were used to analyze cell viability.

### Statistics.

Statistical significance was determined by 2-tailed Student’s unpaired *t* test. The significance among multiple groups was determined by 1-way or 2-way ANOVA by GraphPad Prism Version 6. Tukey’s multiple-comparison test was used as post hoc test. The *P* values of GSEA analysis were calculated by using Fisher’s exact test. Pearson’s correlation test on log_10_- transformed data was performed by using GraphPad Prism Version 6. *P* < 0.05 was considered statistically significant.

### Study approval.

This study was approved by Stockholm Regional Ethics Committee and executed in agreement with the Declaration of Helsinki. Informed written and oral consent was obtained from all the research participants. Porcine experiments were performed at MD Biosciences Innovalora Ltd. with local IRB approval. All animal care was in accordance with *Guide for the Care and Use of Laboratory Animals* (National Academies Press, 2011) and UCLA IACUC (UCLA protocol no. 16–018).

### Data availability.

Human wound healing RNA-Seq data are publicly available (GSE174661, GSE196773) (50). Data from *PPIF-*KD microarray experiments (E-MTAB-13920) can be accessed through the relevant databases. Supporting data for figures can be found in the Supporting Data Values file.

## Author contributions

RB, JDW, EBW, and OSS conceptualized the project and designed the experiments. RB, MT, MH, MM, MC, EPT, CS, and NW performed experiments and analyzed data. All authors contributed to the manuscript writing, which was led by RB and JDW.

## Supplementary Material

Supplemental data

Unedited blot and gel images

Supporting data values

## Figures and Tables

**Table 3:**
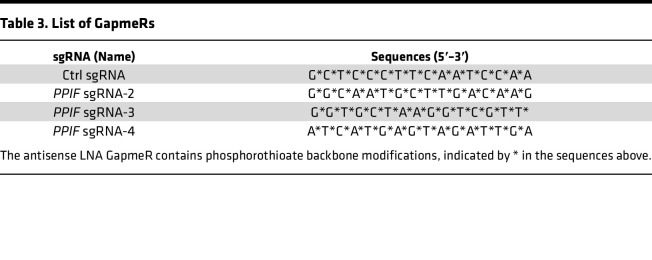
List of GapmeRs

**Table 2:**
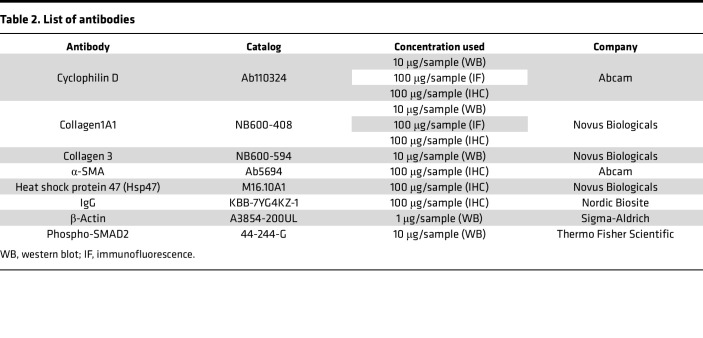
List of antibodies

**Table 1:**
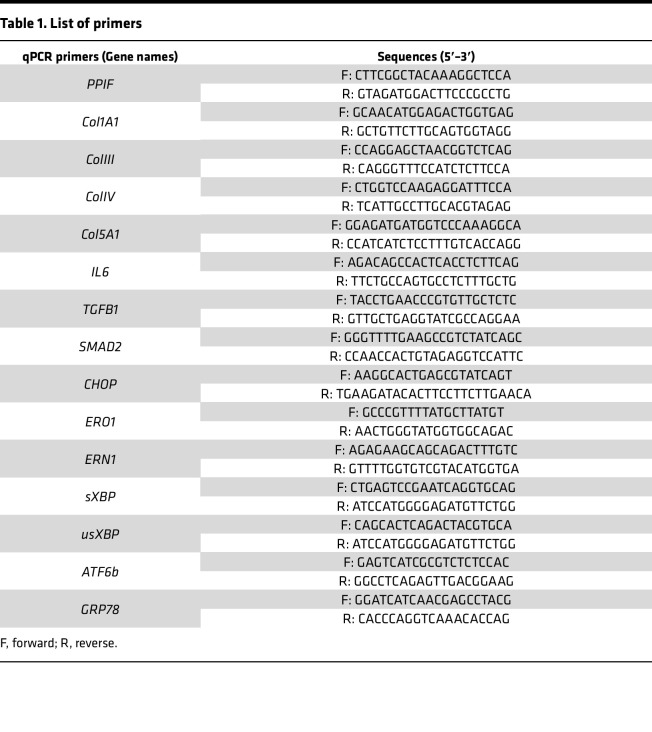
List of primers

## Version 1 04/02/2024

In-Press Preview

## Version 2 05/08/2024

Electronic publication
