# Supplementary material for: Role of the mitochondrial protein cyclophilin D in skin wound healing and collagen secretion
Source: JCI Insight. 2024 Apr 2;9(9):e169213. doi: 10.1172/jci.insight.169213 (PMC11141914; doi:10.1172/jci.insight.169213)
Supplement: Supplemental data [file jciinsight-9-169213-s215.pdf]

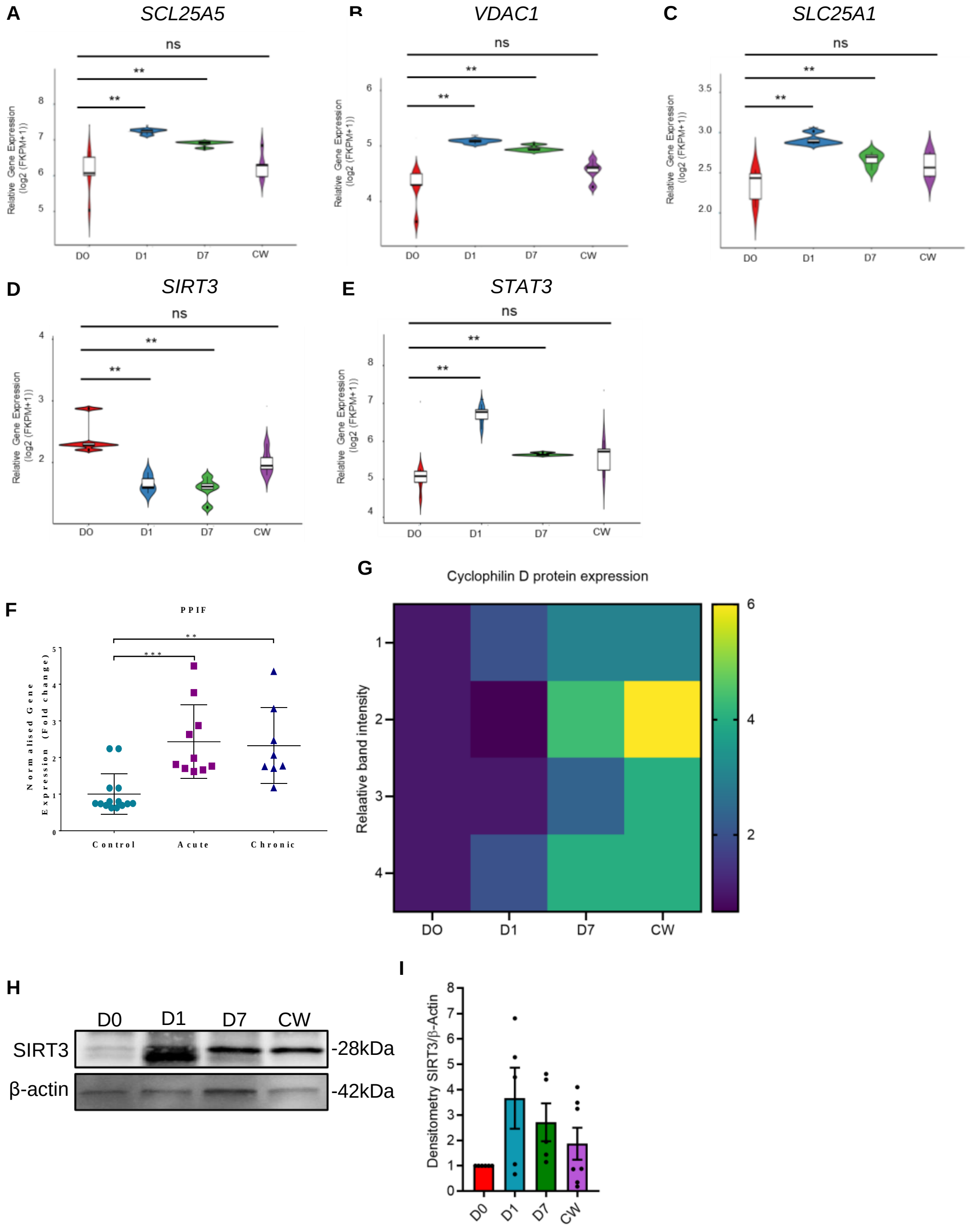

Supplemental Figure 1. Cyclophilin D is upregulated in re-epithelization (day 1) and granulation tissue phase (day 7) of normal wound healing and chronic venous leg wounds. (**A-E**) Publicly available RNA seq gene expression analysis of mPTP genes *SLC25A5*, *VDAC1*, SLC25A51, *SIRT3* and *STAT3* in intact skin biopsies, wound biopsies and CW biopsies. (**F**) RT-qPCR analysis (mean ± SEM) of *PPIF* transcript expression in 7 intact skin biopsies, 10 acute wound biopsies and 8 CW biopsies. One-way ANOVA. (**G**) Heat map representation of *PPIF* expression in 3 independent western blot experiments. N = 3. (**H**) Representative immunoblot and (**I**) quantification (mean ± SEM) of SIRT3 protein levels in acute and chronic wound biopsies. *** = $p < 0.0001$; ** = $p < 0.005$.

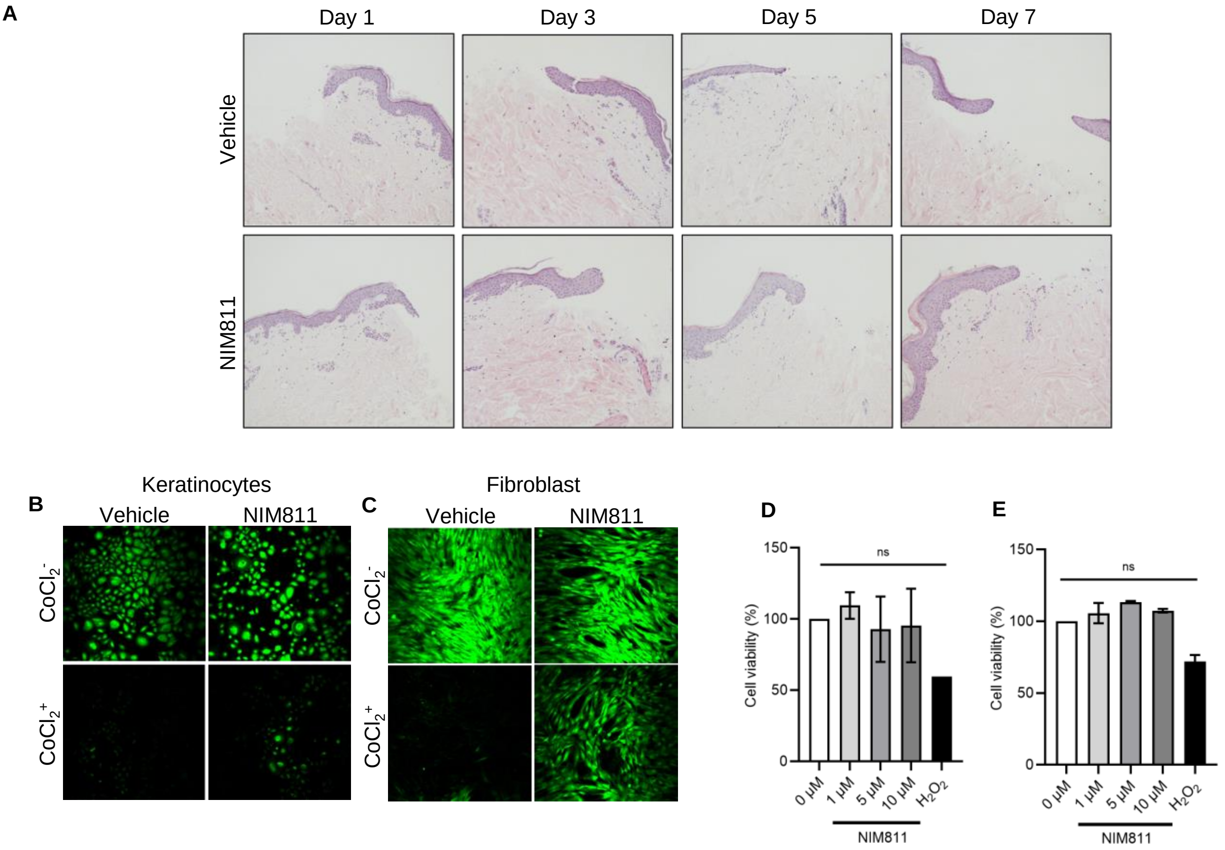

Supplemental Figure 2. NIM811 successfully inhibits Cyclophilin D. (**A**) Representative H&E-stained vehicle or NIM811-treated ex vivo human explant wounds. (**B-C**) Representative confocal microscopy images of calcein fluorescence before and after the addition of cobalt chloride ($CoCl_2^-$) with vehicle and NIM811-treated (**B**) keratinocytes and (**C**) fibroblasts**.** Note that this control experiment shows that when Cyclophilin D/PPIF is inhibited, some calcein fluorescence remains as opposed to when the permeability transition pore is more open. (**D-E**) Quantification (mean ± SEM) of viability in (**D**) keratinocytes and (**E**) fibroblasts. N = 3 biological replicates; unpaired t-test.

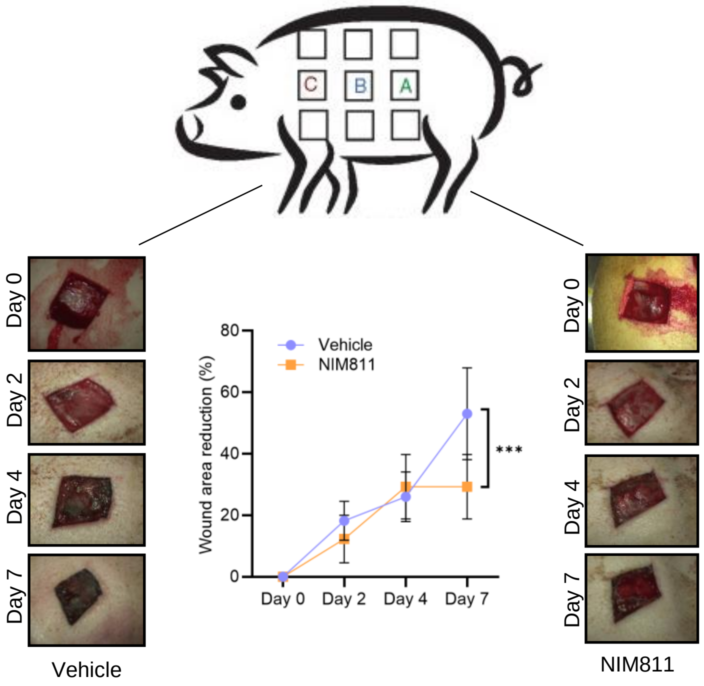

Supplemental Figure 3. Cyclophilin D/PPIF specific inhibitor NIM811 impairs porcine wound closure. Representative wound images in porcine wound model and photographs depicting the impact of NIM811 *vs.* vehicle treatment on wound reduction area quantification (mean ± SEM). Percentage of healing was determined by the following calculation: wound area - initial wound area / initial wound area *100. N = 5 biological replicates; one-way ANOVA. *** = $p < 0.001$.

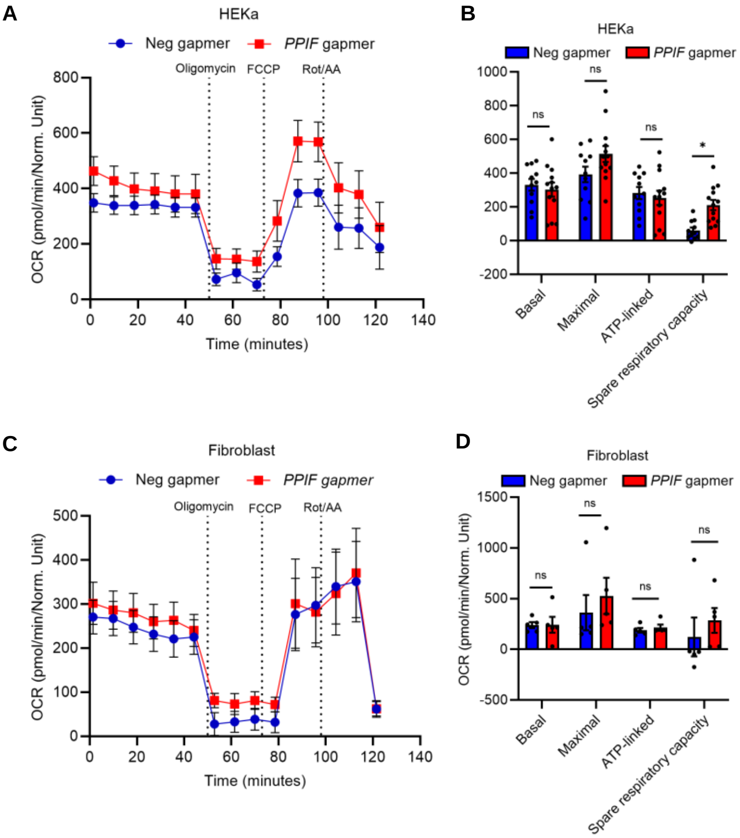

Supplemental Figure 4. Cyclophilin D downregulation does not affect mitochondrial function in keratinocytes or fibroblasts. (**A**) Oxygen consumption rate (mean ± SEM) of *PPIF* knockdown keratinocytes, and (**B**) quantification (mean ± SEM) of oxidative respiration parameters. (**C**) Oxygen consumption rate (mean ± SEM) of *PPIF* knockdown fibroblasts, and (**D**) quantification (mean ± SEM) of oxidative respiration parameters. N = 3 biological replicates. One-way ANOVA. Ns = non-significant.

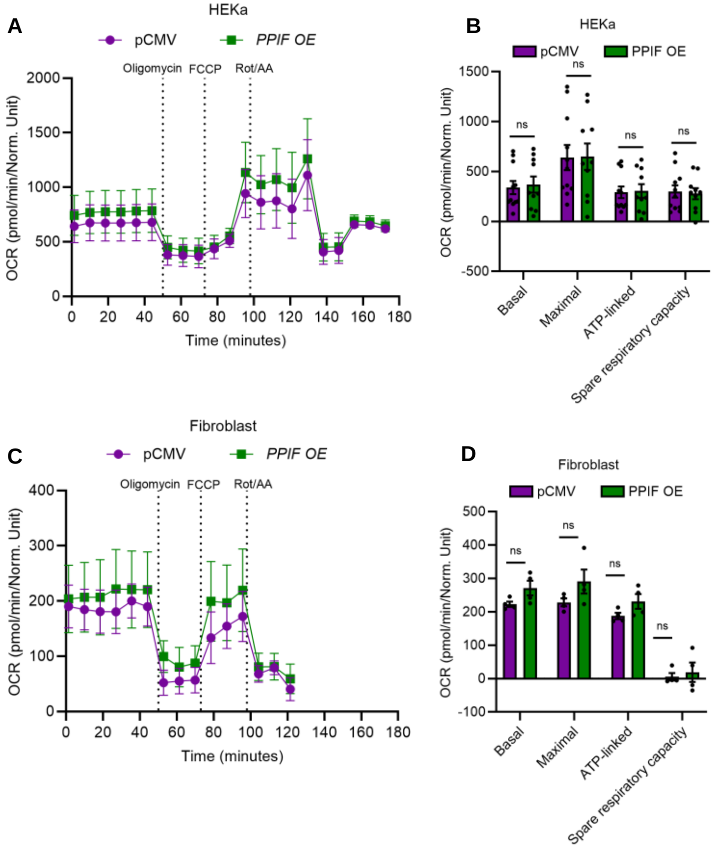

Supplemental Fig 5. Cyclophilin D overexpression does not affect mitochondrial function in keratinocytes or fibroblasts. (**A**) Oxygen consumption rate (mean ± SEM) of PPIF overexpression keratinocytes, and (**B**) quantification (mean ± SEM) of oxidative respiration parameters. (**C**) Oxygen consumption rate (mean ± SEM) of PPIF overexpression fibroblasts, and (**D**) quantification (mean ± SEM) of oxidative respiration parameters. N = 3 biological replicates. One-way ANOVA. Ns = non-significant.

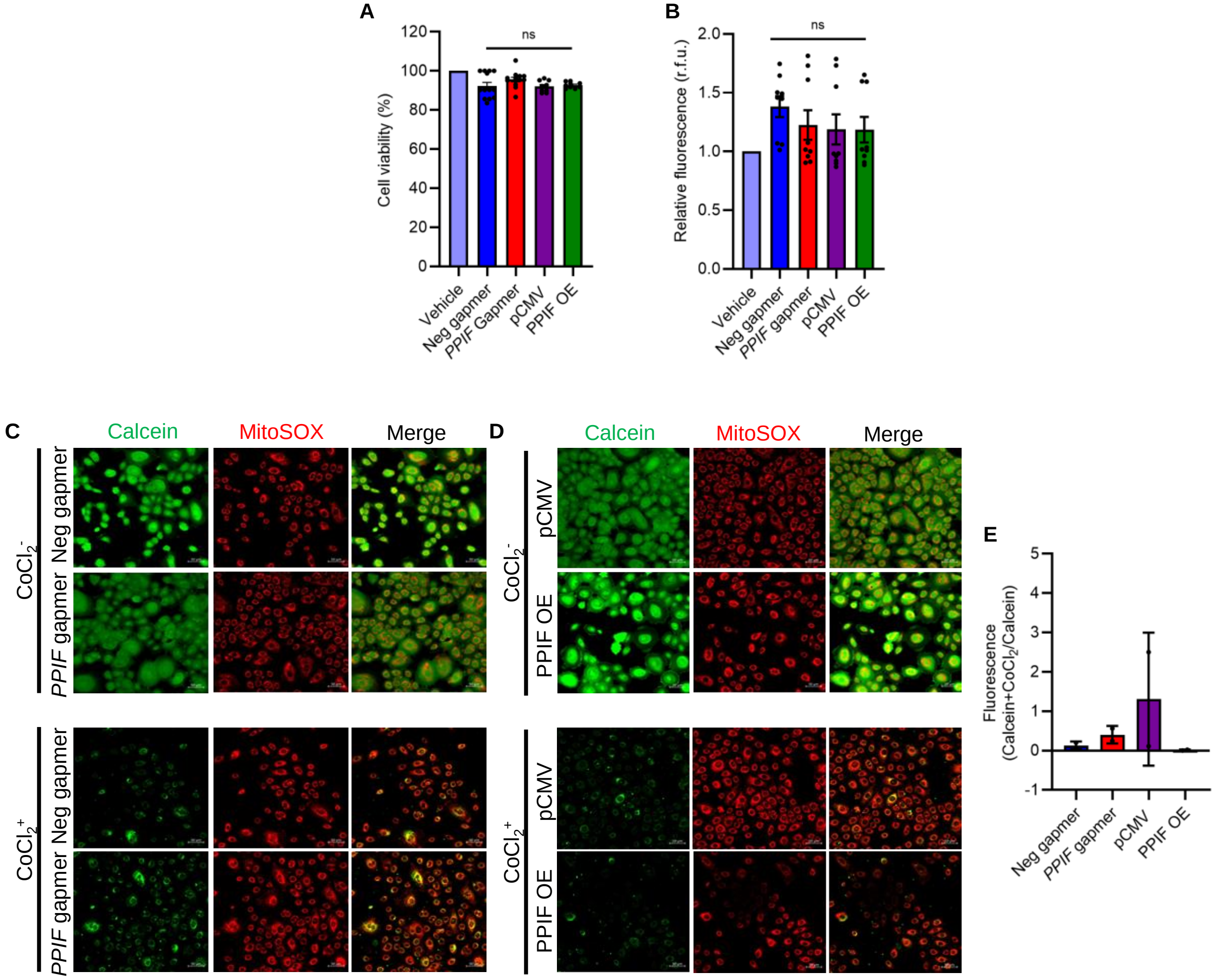

Supplemental Figure 6. Cyclophilin D KD or OE does not affect cell viability or superoxide production in keratinocytes but inhibits and enhances, respectively, mPTP activity in keratinocytes. (**A**) Cell viability measured with presto blue assay for *PPIF* KD and OE keratinocytes. N = 3 biological repeats. One-way ANOVA. (**B**) Superoxide production measured with MitoSOX Red. Data represent one of two (n = 2) independent experiments showing a similar pattern. One-way ANOVA. (**C-D**) Mitochondrial permeability transition pore (mPTP) calcein quenching assay measured by fluorescence microscopy for (**C**) *PPIF* KD and (**D**) OE keratinocytes. Scale bar = 50 µm. (**E**) Quantification (mean ± SD) of the mPTP assay based on the fluorescence intensity before and after adding cobalt chloride. Data represent one of three (n = 3) independent experiments showing a similar staining pattern. 2-way ANOVA.

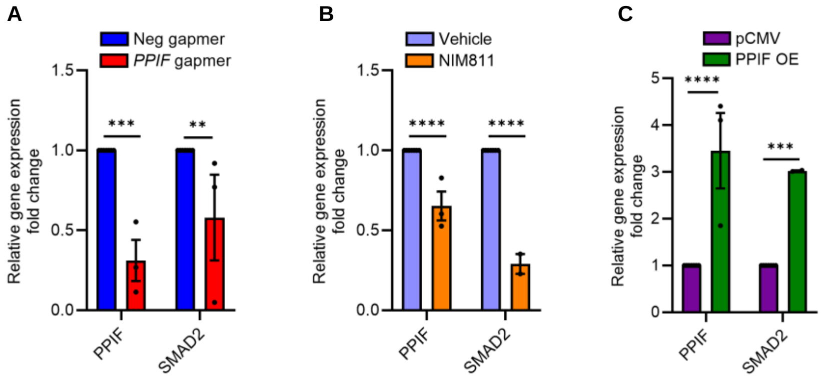

Supplemental Figure 7. Cyclophilin D knockdown and overexpression inhibits and enhances, respectively *SMAD2* expression in keratinocytes. (**A-C**) Relative mRNA expression (mean ± SEM) of the downstream TGF-beta signaling protein *SMAD2* and *PPIF* in (**A**) *PPIF* gapmer, (**B**) NIM811-treated, and (**C**) PPIF OE keratinocytes. Note that there is decreased expression of SMAD2 with *PPIF* KD signifying the correlation with microarray pathways and the importance of TGF beta signaling in keratinocytes. N = 3; 2-tailed, unpaired t-test.

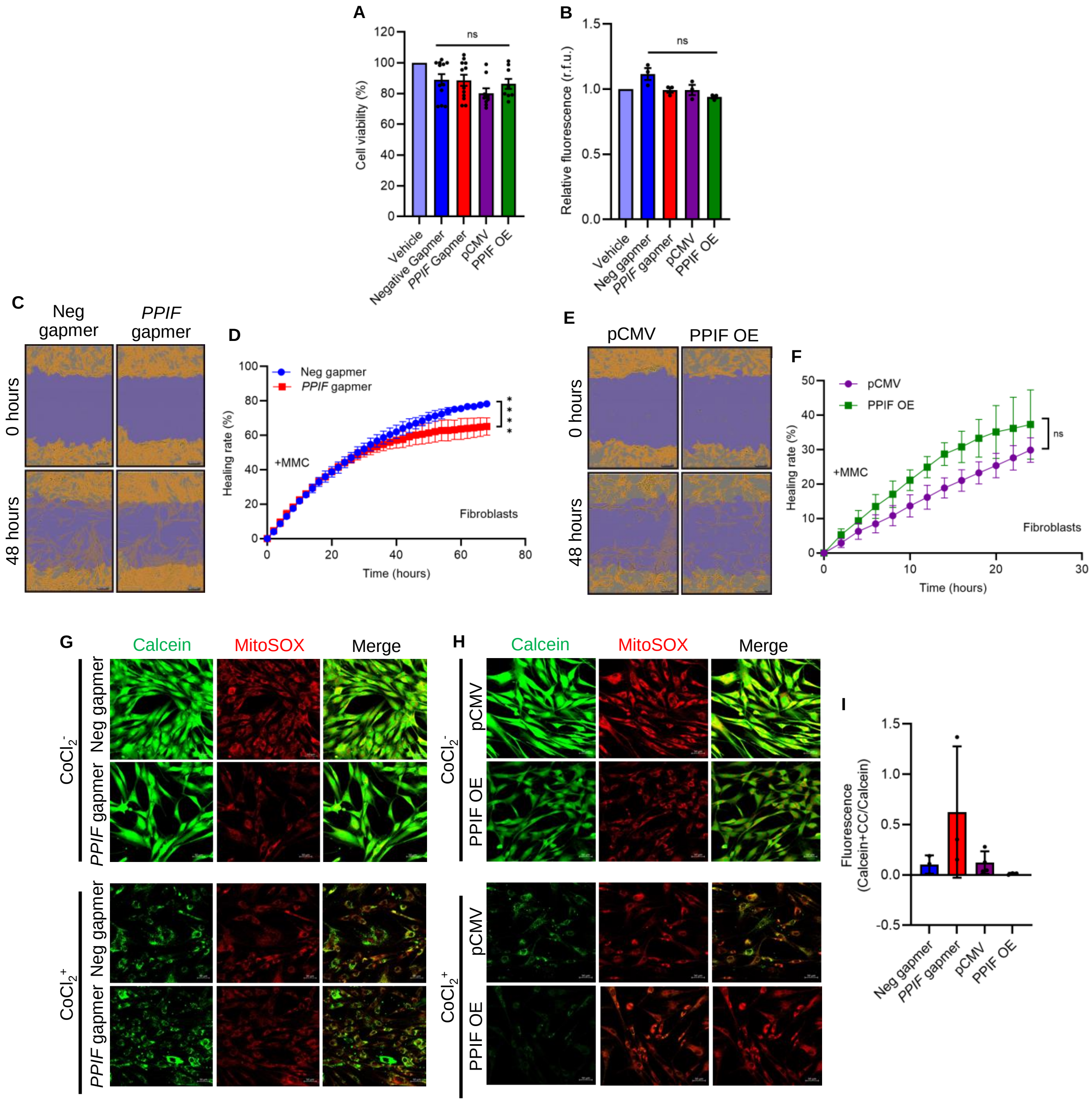

Supplemental Figure 8. Cyclophilin D knockdown and overexpression does not affect cell viability, superoxide production or migration but increases mPTP activity in fibroblasts. (**A**) Cell viability (mean ± SEM) of *PPIF* KD and OE fibroblasts. N = 3 biological replicates. One-way ANOVA. (**B**) Superoxide production measured with mitoSOX Red. Data represent one of two independent experiments showing a similar pattern. (**C**) Representative images and (**D**) quantification (mean ± SEM) of scratch assay in *PPIF* KD fibroblasts treated with mitomycin C. N = 3 biological repliates. One-way ANOVA. (**E**) Representative images and (**F**) quantification (mean ± SEM) of scratch assay in pCMV and PPIF OE fibroblasts treated with mitomycin C. Two-way ANOVA. (**G**) mPTP calcein quenching assay measured by fluorescence microscopy for *PPIF* KD and (**H**) OE fibroblasts. Scale bar = 50 µm. (**I**) Quantification (mean ± SEM) mPTP assay. N = 3 biological replicates; One-way ANOVA. **** = $p < 0.0001$; * = $p < 0.05$.

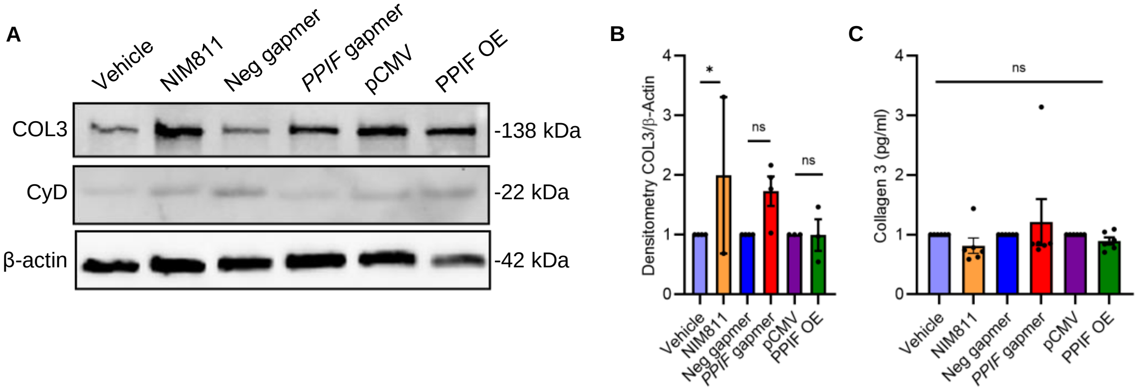

Supplemental Figure 9. Cyclophilin D levels do not impact collagen 3 expression and secretion in human dermal fibroblasts. (**A**) Representative immunoblot and (**B**) densitometric quantification (mean ± SD) of collagen 3 in fibroblast lysates. (**C**) Collagen 3 secretion (mean ± SEM) to media by fibroblasts measured by ELISA. Data represent one of two (n = 2) independent experiments showing a similar pattern. One-way ANOVA. * = $p < 0.05$.

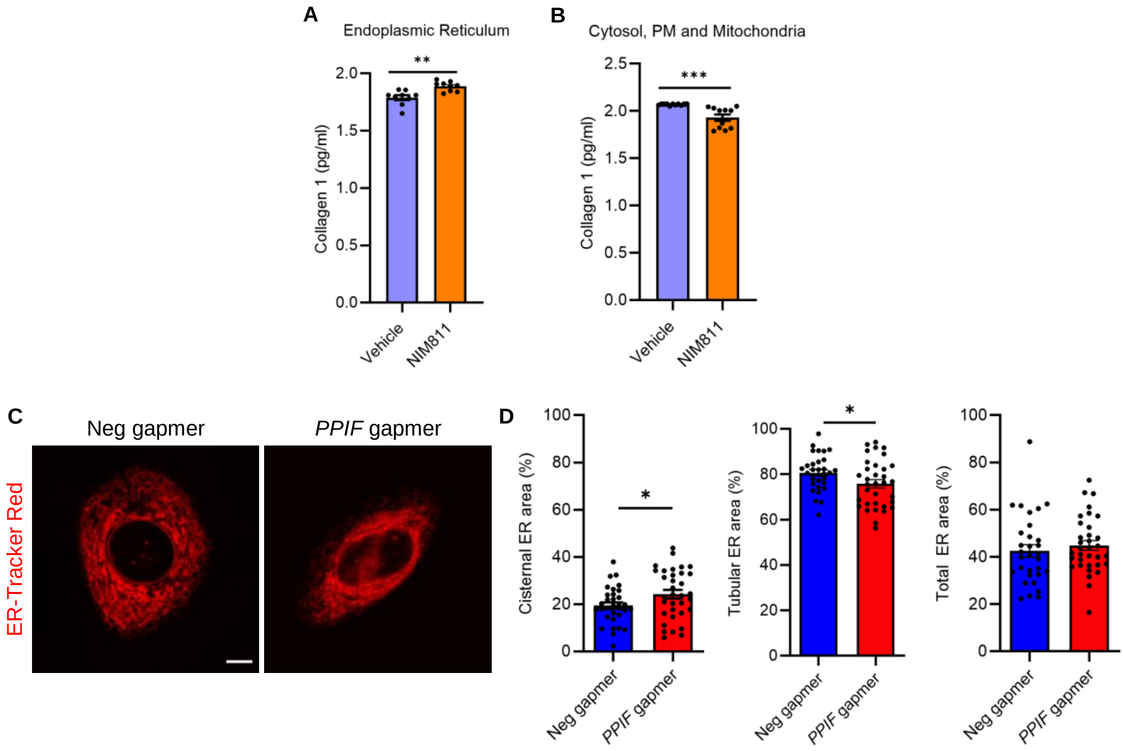

Supplemental Figure 10. Cyclophilin D inhibition increases collagen accumulation in ER. (**A-B**) Quantification (mean ± SEM) of collagen 1 accumulation in (**A**) ER as well as (**B**) cytosol, plasma membrane, and mitochondrial isolates in NIM811-treated fibroblasts measured by ELISA. N = 2 biological repeats. (**C**) Representative immunofluorescence images of ER morphology in negative and *PPIF* gapmer HEKa. (**D**) Quantification (mean ± SEM) of cisternal, tubular, and total ER area. N = 3 biological replicates. 2-tailed, unpaired t-test. * = p<0.05.

Supplemental information

Supplemental methods

RNA *in situ* hybridization

Briefly, sections were baked at 60°C for 1 hour before dewaxing, rehydration, and pre-treatment. Next, sections were hybridized with RNAScope probe hs-PPIF (accession number NM_005729), alongside positive control probe Hs-PPIB (313910, accession number NM_000942.4) and negative control probe dapB (310043, accession number EF191515), with detection of probe binding sites performed with RNAScope 2.5 HD Reagent kit – Red (Advanced Cell Diagnostics). Probe signal was amplified through a series of amplification steps before sections were counterstained with 50 % Gill no. 1 haematoxylin (Sigma-Aldrich) for 2 minutes at RT and blued with 0.002 % ammonia water (Thermo Fischer Scientific). Sections were then mounted with Ecomount (Biocare Medical) and stored at RT until imaging.

Immunofluorescence

Antigen retrieval was performed in a pressure cooker using 10 mM sodium citrate pH 6.0 for 7 minutes. After antigen retrieval, sections were blocked with 10 % normal donkey serum (NDS) for 1 hour then incubated with PPIF (Abcam) primary antibody (1:250) for 1 hour at RT. After washing with tris-buffered saline with Tween 20 (TBST), sections were incubated with Alexa-fluor 555 Goat anti-donkey secondary antibody (1:1000) (Abcam) for 1 hour at RT before staining with DAPI (1:2000) (Thermo Fischer Scientific) for 5 minutes at RT. Finally, sections were mounted using Ecomount (Biocare Medical) and stored at 4 °C until imaging.

Human skin explant ex vivo model

Human skin wound explants were obtained from 8 healthy patients, washed in phosphate buffer solution (PBS, Sigma-Aldrich) and cultured in DMEM supplemented with 10 % FBS (Thermo Fischer Scientific), glucose and Pen Strep (100 units/mL Penicillin and 100 µg/mL Streptomycin) (Thermo Fisher Scientific) with three to five explants from three independent donors used for each treatment. An excisional 2 mm wound punch was made on the epidermis of the skin followed by 6 mm biopsy punch for the excision of the skin. The excised skin was cultured in a 24 well plate with media containing vehicle (0.1 % DMSO) (Sigma-Aldrich) or 5 µM NIM811 (MedChemExpress) at 37 ℃ in 5 % CO_2. This transdermal treatment containing media was replaced every other day following injury and wound samples were collected for histological staining and RT-qPCR analysis on days 0, 1, 3, 5, and 7 after the wound induction. These biopsies were fixed in PFA (Sigma-Aldrich) for 24 hours followed by their transfer in 70 % ethanol (Sigma-Aldrich) for histological sectioning and sent for histological analysis at the indicated time points.

Immunohistochemistry

The sections were heated at 60 °C for 10 minutes and washed with xylene (2x for 10 minutes) and 90 % ethanol (2x for 10 minutes) for deparaffinization. Antigen retrieval and primary antibody incubation was carried out in a hydration chamber at RT for 1 hour. Two sections of each sample were incubated with primary antibody and one section was incubated with IgG only as negative control. Counterstaining was done with H&E. Images were acquired using Zeiss Axio Scan.Z1 digital slide scanner.

Immunoblotting

Cell pellet obtained from treated cells was resuspended in RIPA buffer (Thermo Fisher Scientific) and kept on ice for 30 minutes with the inversion at every 10-minute

interval. Resuspended cells were centrifuged at 13000 g for 10 minutes and the cell supernatant was used for western blotting. Isolated subcellular fractions (50 ng) were loaded on 4-20 % gradient SDS polyacrylamide gels (Biorad) and ran at 80 V followed by 120 V for 1-1.5 hours. Gel was transferred onto nitrocellulose membrane and blocked in 5 % skim milk (Thermo Fisher Scientific) followed by incubation with primary antibody (1:1000) at 4 °C overnight with slow shaking. Next day, membranes were washed with TBST (Tris Buffered Saline with 0.05 % Tween 20) three times, incubated with HRP-conjugated secondary antibody for 1 hour and finally detected with femto ECL solution (Thermo Fisher Scientific). The primary antibodies used were: CyD (Abcam), Collagen 1 (Novus biologicals), Collagen 3 (Novus biologicals) and α-SMA (Abcam). The secondary antibodies, Anti-mouse or Anti-rabbit, were used at 1:15000 dilution.

Supplemental figure legends

Supplemental Figure 1. Cyclophilin D is upregulated in re-epithelization (day 1) and granulation tissue phase (day 7) of normal wound healing and chronic venous leg wounds. (**A-E**) Publicly available RNA seq gene expression analysis of mPTP genes *SLC25A5*, *VDAC1*, SLC25*A51, SIRT3* and *STAT3* in intact skin biopsies, wound biopsies and CW biopsies. (**F**) RT-qPCR analysis (mean ± SEM) of *PPIF* transcript expression in 7 intact skin biopsies, 10 acute wound biopsies and 8 CW biopsies. One-way ANOVA. (**G**) Heat map representation of *PPIF* expression in 3 independent western blot experiments. N = 3. (**H**) Representative immunoblot and (**I**) quantification (mean ± SEM) of SIRT3 protein levels in acute and chronic wound biopsies. *** = $p < 0.0001$; ** = $p < 0.005$.

Supplemental Figure 2. NIM811 successfully inhibits Cyclophilin D. (**A**) Representative H&E-stained vehicle or NIM811-treated ex vivo human explant wounds. (**B-C**) Representative confocal microscopy images of calcein fluorescence before and after the addition of cobalt chloride ($CoCl_2^-$) with vehicle and NIM811-treated (**B**) keratinocytes and (**C**) fibroblasts**.** Note that this control experiment shows that when Cyclophilin D/PPIF is inhibited, some calcein fluorescence remains as opposed to when the permeability transition pore is more open. (**D-E**) Quantification (mean ± SEM) of viability in (**D**) keratinocytes and (**E**) fibroblasts. N = 3 biological replicates; unpaired t-test.

Supplemental Figure 3. Cyclophilin D/PPIF specific inhibitor NIM811 impairs porcine wound closure. Representative wound images in porcine wound model and photographs depicting the impact of NIM811 *vs.* vehicle treatment on wound reduction area quantification (mean ± SEM). Percentage of healing was determined by the following calculation: wound area - initial wound area / initial wound area *100. N = 5 biological replicates; one-way ANOVA. *** = $p < 0.001$.

Supplemental Figure 4. Cyclophilin D downregulation does not affect mitochondrial function in keratinocytes or fibroblasts. (**A**) Oxygen consumption rate (mean ± SEM) of *PPIF* knockdown keratinocytes, and (**B**) quantification (mean ± SEM) of oxidative respiration parameters. (**C**) Oxygen consumption rate (mean ± SEM) of *PPIF* knockdown fibroblasts, and (**D**) quantification (mean ± SEM) of oxidative respiration parameters. N = 3 biological replicates. One-way ANOVA. Ns = non-significant.

Supplemental Fig 5. Cyclophilin D overexpression does not affect mitochondrial function in keratinocytes or fibroblasts. (**A**) Oxygen consumption rate (mean ± SEM) of PPIF overexpression keratinocytes, and (**B**) quantification (mean ± SEM) of oxidative respiration parameters. (**C**) Oxygen consumption rate (mean ± SEM) of PPIF overexpression fibroblasts, and (**D**) quantification (mean ± SEM) of oxidative respiration parameters. N = 3 biological replicates. One-way ANOVA. Ns = non-significant.

Supplemental Figure 6. Cyclophilin D KD or OE does not affect cell viability or superoxide production in keratinocytes but inhibits and enhances,

respectively, mPTP activity in keratinocytes. (**A**) Cell viability measured with presto blue assay for *PPIF* KD and OE keratinocytes. N = 3 biological repeats. One-way ANOVA. (**B**) Superoxide production measured with MitoSOX Red. Data represent one of two (n = 2) independent experiments showing a similar pattern. One-way ANOVA. (**C-D**) Mitochondrial permeability transition pore (mPTP) calcein quenching assay measured by fluorescence microscopy for (**C**) *PPIF* KD and (**D**) OE keratinocytes. Scale bar = 50 µm. (**E**) Quantification (mean ± SD) of the mPTP assay based on the fluorescence intensity before and after adding cobalt chloride. Data represent one of three (n = 3) independent experiments showing a similar staining pattern. 2-way ANOVA.

Supplemental Figure 7. Cyclophilin D knockdown and overexpression inhibits and enhances, respectively *SMAD2* expression in keratinocytes. (**A-C**) Relative mRNA expression (mean ± SEM) of the downstream TGF-beta signaling protein *SMAD2* and *PPIF* in (**A**) *PPIF* gapmer, (**B**) NIM811-treated, and (**C**) PPIF OE keratinocytes. Note that there is decreased expression of SMAD2 with *PPIF* KD signifying the correlation with microarray pathways and the importance of TGF beta signaling in keratinocytes. N = 3; 2-tailed, unpaired t-test.

Supplemental Figure 8. Cyclophilin D knockdown and overexpression does not affect cell viability, superoxide production or migration but increases mPTP activity in fibroblasts. (**A**) Cell viability (mean ± SEM) of *PPIF* KD and OE fibroblasts. N = 3 biological replicates. One-way ANOVA. (**B**) Superoxide production measured with mitoSOX Red. Data represent one of two independent experiments showing a similar pattern. (**C**) Representative images and (**D**) quantification (mean ± SEM) of scratch assay in *PPIF* KD fibroblasts treated with mitomycin C. N = 3 biological repliates. One-way ANOVA. (**E**) Representative images and (**F**) quantification (mean ± SEM) of scratch assay in pCMV and PPIF OE fibroblasts treated with mitomycin C. Two-way ANOVA. (**G**) mPTP calcein quenching assay measured by fluorescence microscopy for *PPIF* KD and (**H**) OE fibroblasts. Scale bar = 50 µm. (**I**) Quantification (mean ± SEM) mPTP assay. N = 3 biological replicates; One-way ANOVA. **** = $p < 0.0001$; * = $p < 0.05$.

Supplemental Figure 9. Cyclophilin D levels do not impact collagen 3 expression and secretion in human dermal fibroblasts. (**A**) Representative immunoblot and (**B**) densitometric quantification (mean ± SD) of collagen 3 in fibroblast lysates. (**C**) Collagen 3 secretion (mean ± SEM) to media by fibroblasts measured by ELISA. Data represent one of two (n = 2) independent experiments showing a similar pattern. One-way ANOVA. * = $p < 0.05$.

Supplemental Figure 10. Cyclophilin D inhibition increases collagen accumulation in ER. (**A-B**) Quantification (mean ± SEM) of collagen 1 accumulation in (**A**) ER as well as (**B**) cytosol, plasma membrane, and mitochondrial isolates in NIM811-treated fibroblasts measured by ELISA. N = 2 biological repeats. (**C**) Representative immunofluorescence images of ER morphology in negative and *PPIF* gapmer HEKa. (**D**) Quantification (mean ± SEM) of cisternal, tubular, and total ER area. N = 3 biological replicates. 2-tailed, unpaired t-test. * = $p<0.05$.
