# Supplementary material for: Role of the mitochondrial protein cyclophilin D in skin wound healing and collagen secretion
Source: JCI Insight. 2024 Apr 2;9(9):e169213. doi: 10.1172/jci.insight.169213 (PMC11141914; doi:10.1172/jci.insight.169213)

Full unedited gel for Figure 1C

Cyclophilin D (PPIF)

With ladder

1 **2** **3**

D0 D1 D7 D0 D1 D7 D0 D1 D7 L **M.Wt (kDa)**

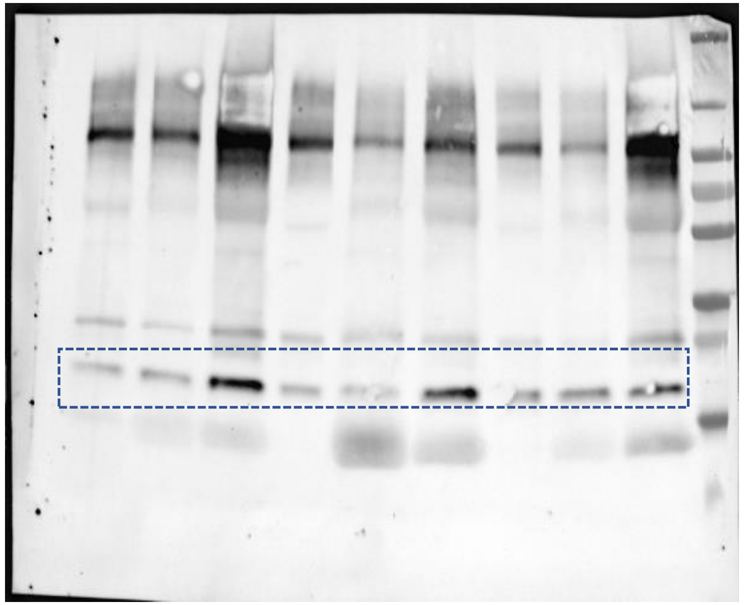

250
130
100
70
55
35
25
15
10

Without ladder

1 **2** **3**

D0 D1 D7 D0 D1 D7 D0 D1 D7

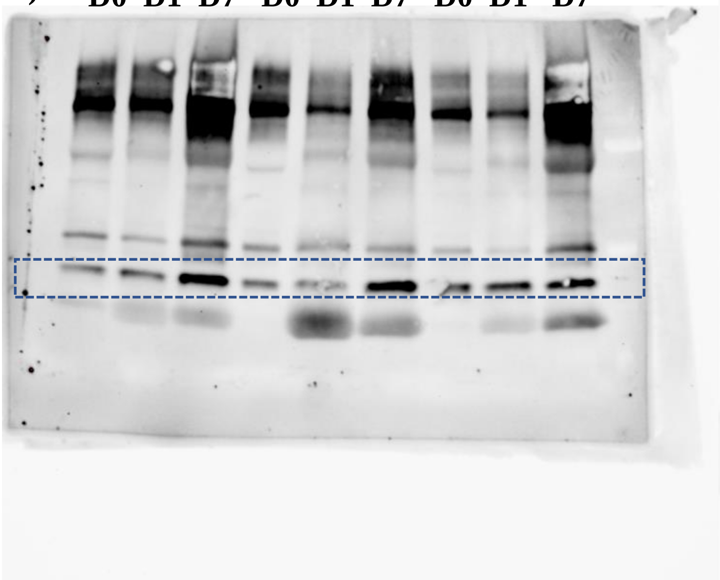

β-actin

1 **2** **3**

D0 D1 D7 D0 D1 D7 D0 D1 D7

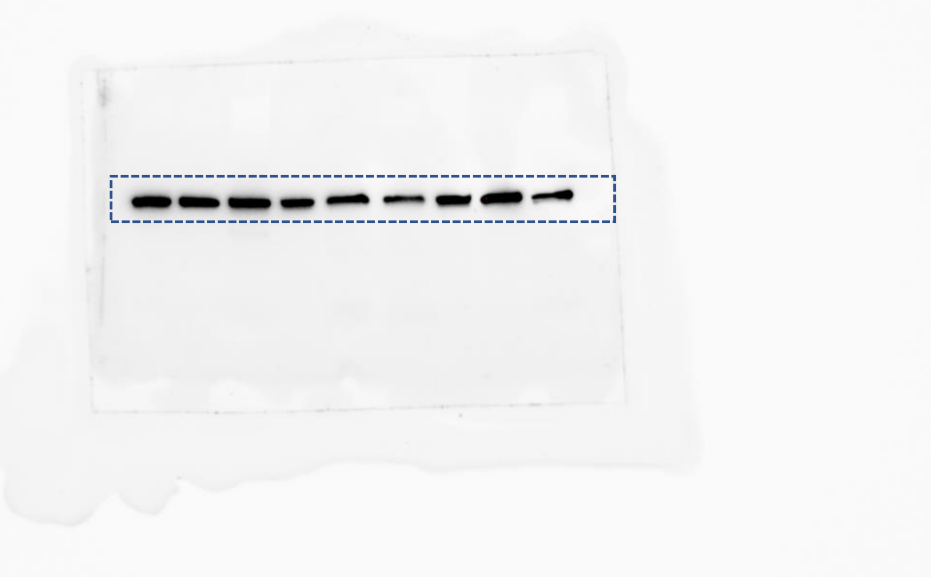

1 **2** **3**

D0 D1 D7 D0 D1 D7 D0 D1 D7

Cyclophilin D
β-actin

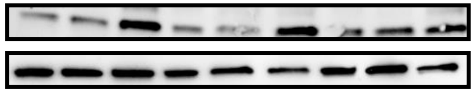

Figure: 1C upper panel

4 **CW**

D0 D1 D7 1 2 3 4 5 L 6

Cyclophilin D
β-actin

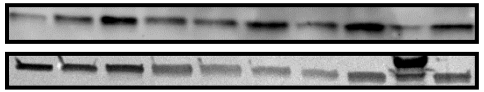

Full unedited gel for Figure 1C

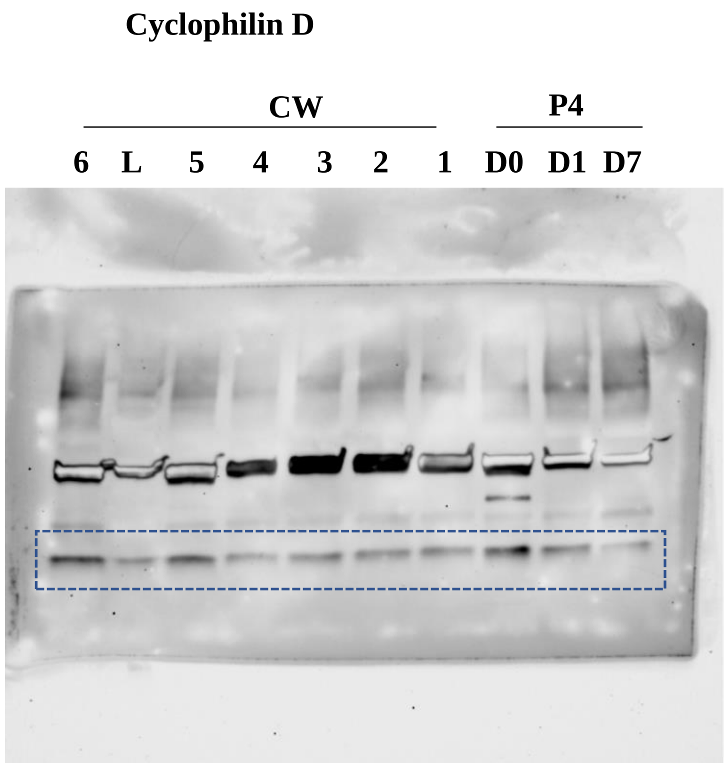

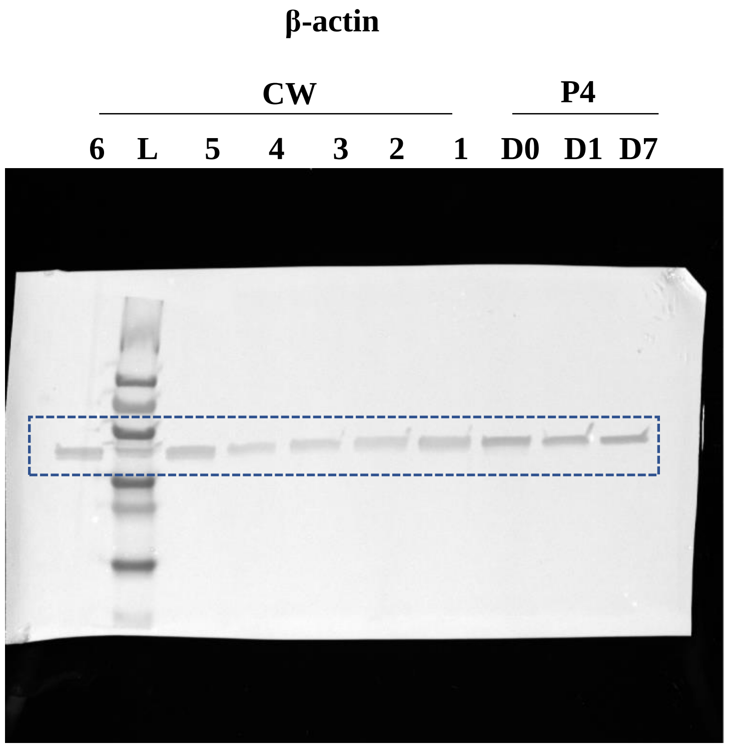

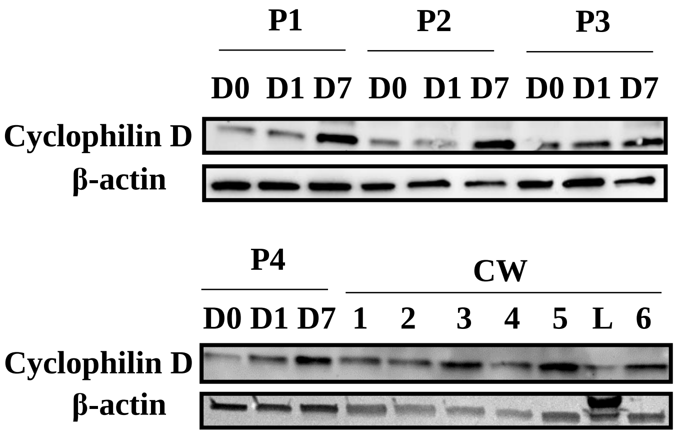

Figure: 1C lower panel

Full unedited gel for Figure 5B

Keratinocytes

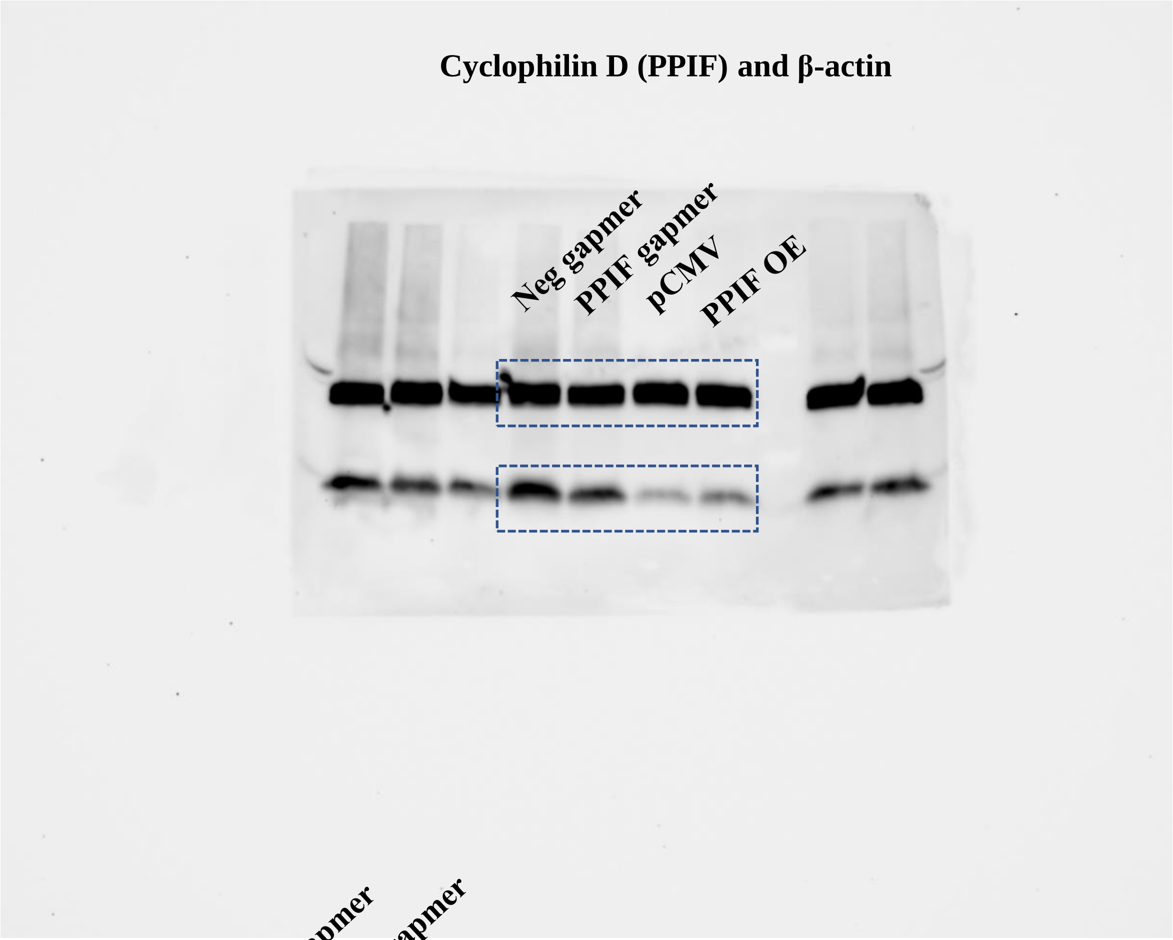

Figure: 5B

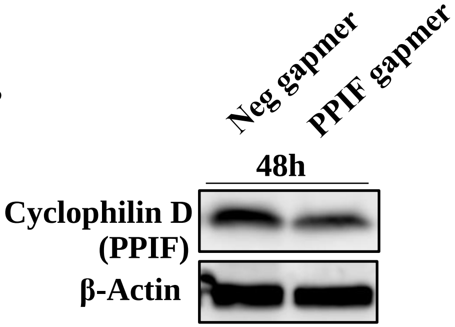

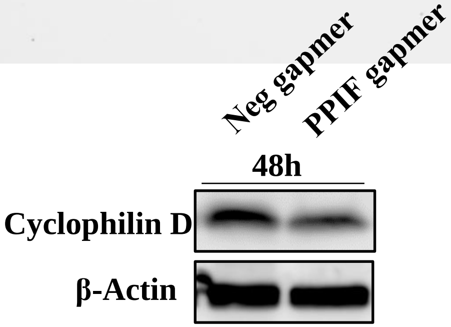

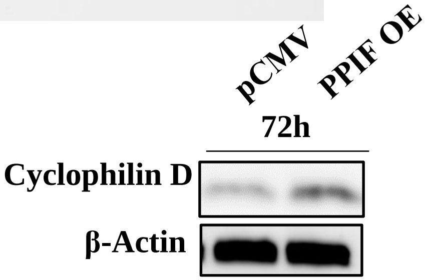

Figure: 5F

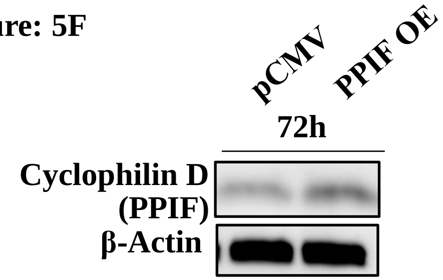

Full unedited gel for Figure 5N

SMAD2-P

Cyclophilin D (PPIF) and β-actin

Keratinocytes

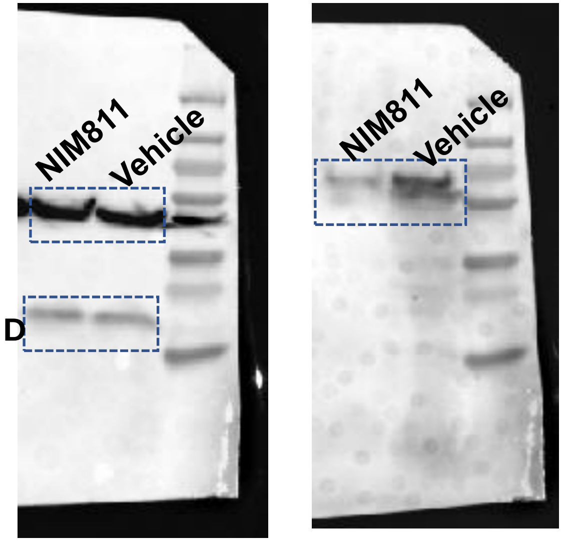

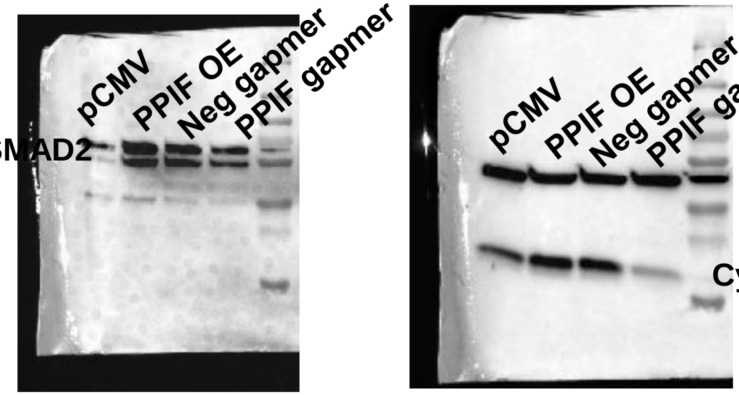

Figure: 5N

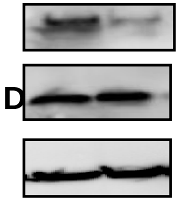

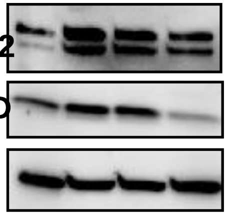

Full unedited gel for Figure 6

Fibroblasts

Beta actin

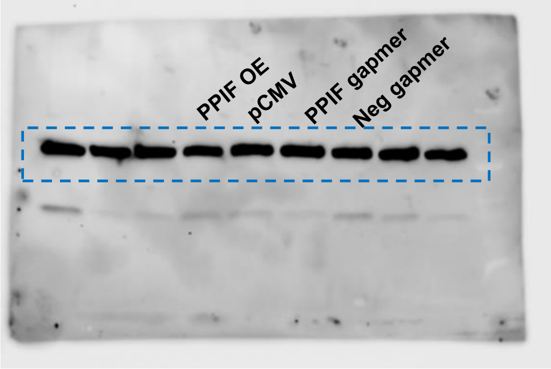

Cyclophilin D (PPIF)
Higher exposure

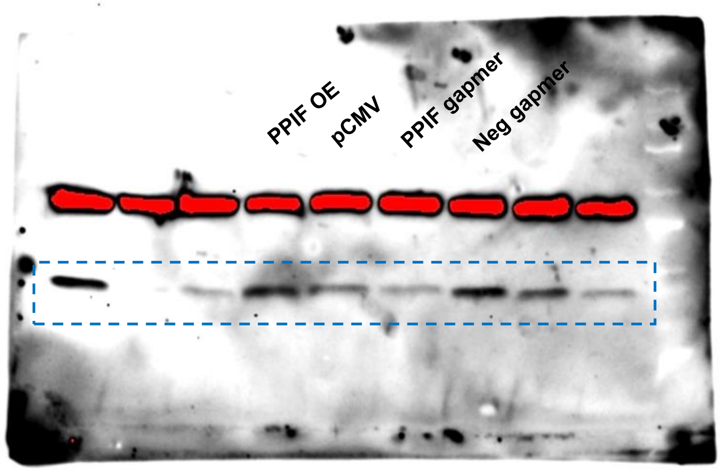

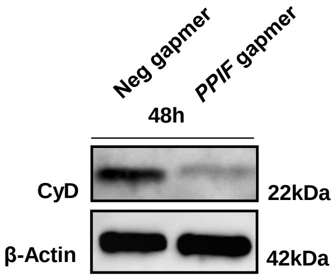

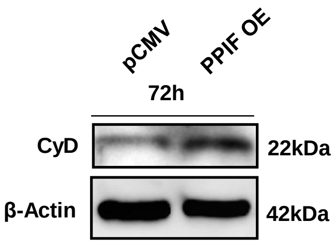

Full unedited gel for Figure 7D

Collagen 1a1 and Collagen 1a2

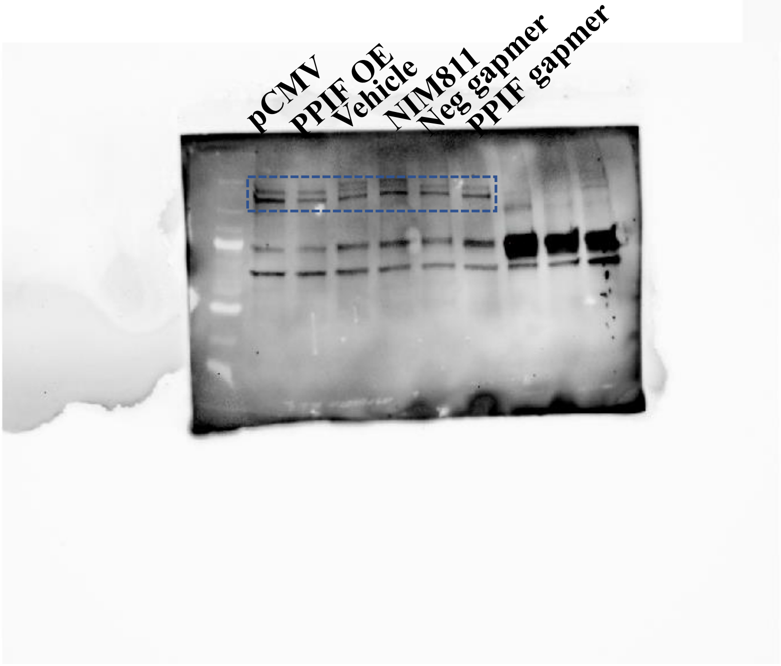

β-actin

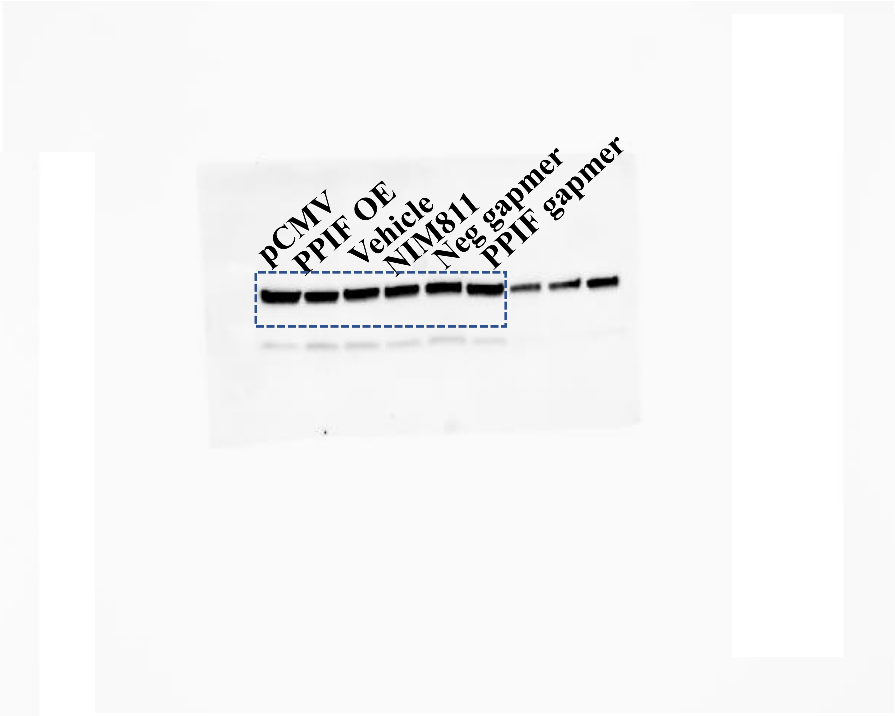

Cyclophilin D (PPIF)

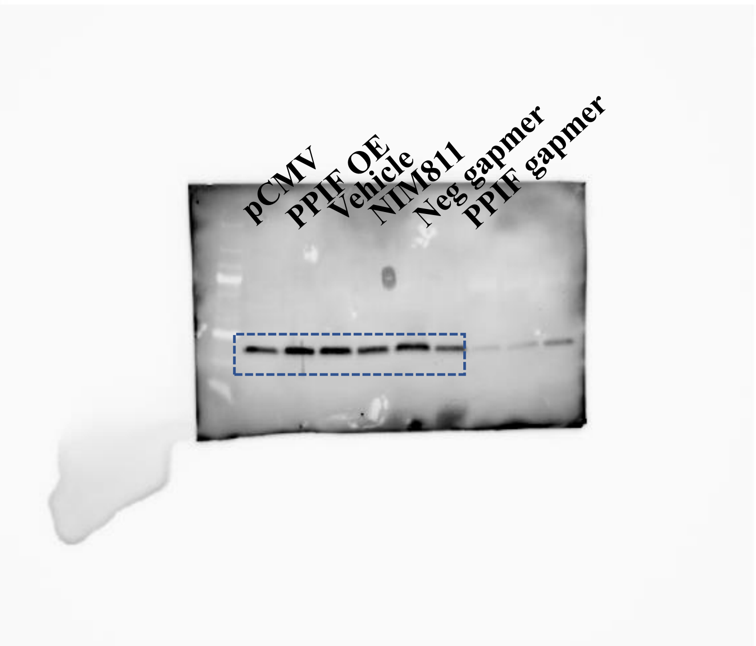

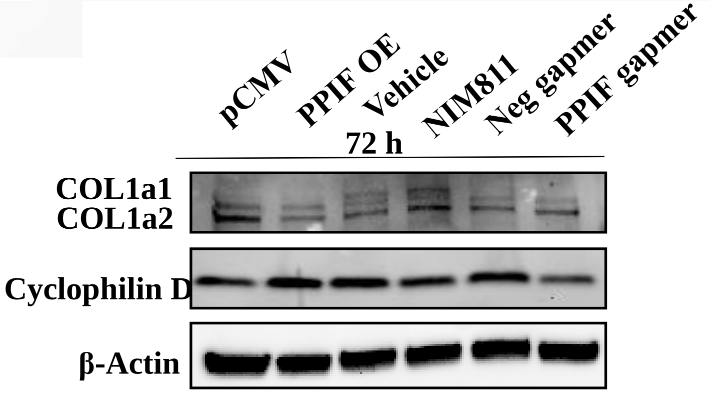

Figure: 7D

Full unedited gel for Supplemental Figure 9A

Collagen III

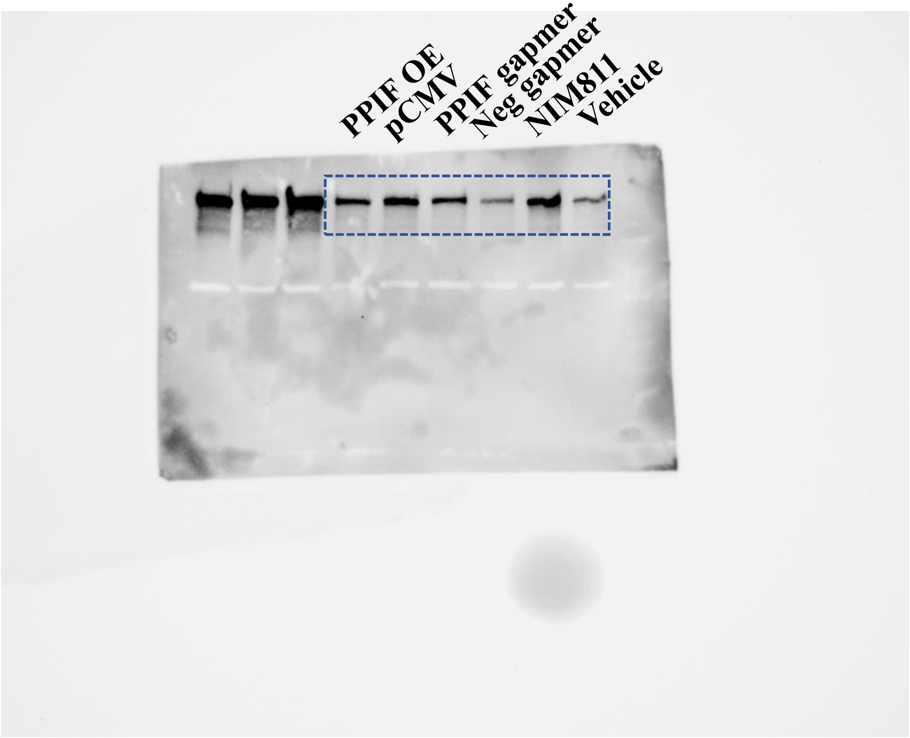

Cyclophilin D (PPIF) and β-actin

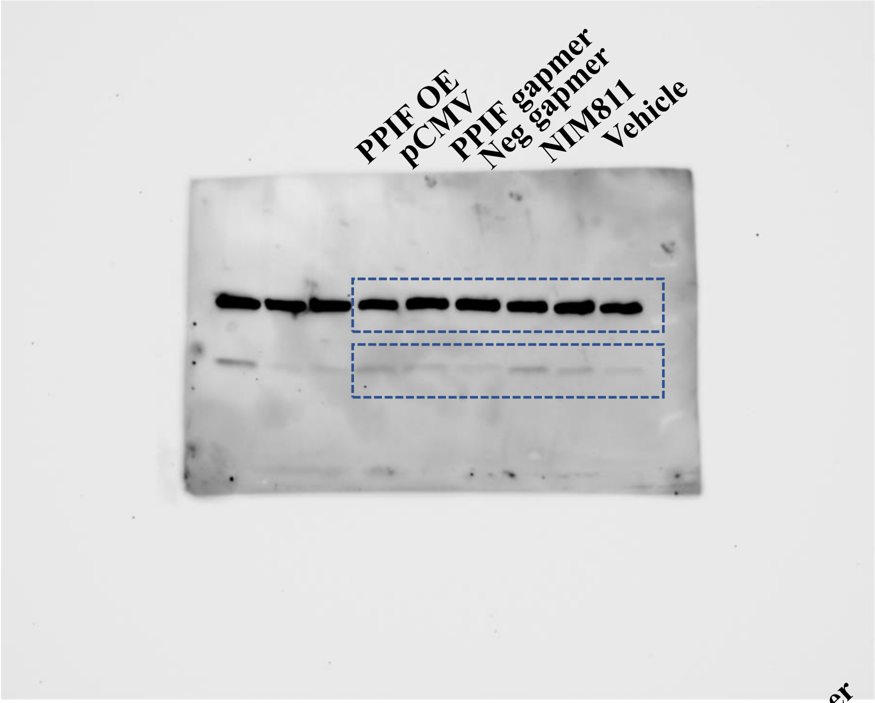

Supplemental Figure: 9a

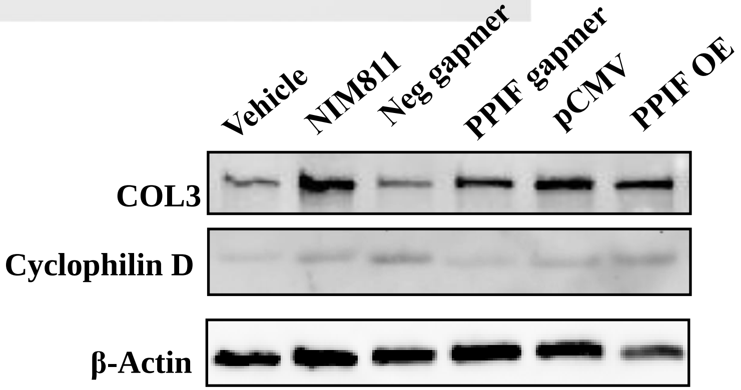

Supplement: Unedited blot and gel images [file jciinsight-9-169213-s216.pdf]
